# Sarcopenia interventions targeted at improving muscle health in adults with cancer: a systematic review and meta-analysis

**DOI:** 10.3389/fnut.2025.1671720

**Published:** 2026-01-05

**Authors:** Yuan Zhao, Liying Ying, Xiaofen Gao, Leiwen Tang, Yuping Zhang, Wanya Pan, Wenhao Tian, Yanjie Liu, Xiuqin Feng

**Affiliations:** 1 Department of Nursing, The Second Affiliated Hospital, Zhejiang University School of Medicine, Hangzhou, Zhejiang, China; 2 Zhejiang University School of Medicine, Hangzhou, Zhejiang, China; 3 Faculty of Nursing, Mahidol University, Bangkok, Thailand

**Keywords:** sarcopenia, cancer survivors, muscle strength, physical functional performance, systematic review, meta-analysis

## Abstract

**Background:**

While evidence is still evolving, sarcopenia interventions show promise as supplemental treatments to mitigate cancer-related muscle loss. It is critical to distinguish this condition from age-related sarcopenia, as cancer-related muscle wasting is driven by an accelerated, multifactorial pathophysiology involving tumor-derived factors, systemic inflammation, and cancer treatments.

**Objectives:**

We aim to ascertain whether sarcopenia interventions are linked to improvements in muscle health among adults with cancer.

**Methods:**

We searched seven databases from 2010 to November 2, 2025. Randomized clinical trials (RCTs) examining the relationship between sarcopenia interventions and at least one of the muscle health indicators (muscle mass, strength, physical performance) were included. We used the Cochrane Risk of Bias Tool 2 Checklist to assess the quality of the evidence. Subgroup analyses were conducted based on intervention type (exercise-only, nutrition-only, multi-component). Additionally, we performed sensitivity analyses and comprehensive publication bias assessments (Egger’s test, funnel plots, and the trim-and-fill method).

**Results:**

Fifty-nine RCTs were included. Meta-analysis showed that sarcopenia interventions were associated with statistically significant improvements in muscle mass (SMD = 0.25; 95% CI, 0.18 to 0.32), muscle strength (SMD = 0.21; 95% CI, 0.15 to 0.26), and some measures of physical performance (6-MWD: SMD = 0.28; 95% CI, 0.15 to 0.42; 30 s sit-to-stand test: SMD = 0.57; 95% CI, 0.35 to 0.78). However, interventions did not significantly improve physical performance measured by SPPB scores (SMD = 0.12; 95% CI, −0.01 to 0.26) or the 5 times chair stand test (SMD = 0.02; 95% CI, −0.15 to 0.18). Subgroup analyses suggested multi-component interventions were most beneficial for muscle mass. Publication bias was detected for some outcomes, but trim-and-fill analyses confirmed the robustness of the overall conclusions for muscle strength and physical performance.

**Conclusion:**

Sarcopenia interventions, particularly multi-component approaches, are associated with statistically significant, though modest, improvements in muscle health in adults with cancer. The clinical relevance of these improvements warrants further investigation. Healthcare professionals should consider integrating these interventions into care plans. Future research should focus on standardizing outcome measurements and optimizing intervention protocols to enhance clinical relevance and impact on quality of life.

**Systematic review registration:**

https://www.crd.york.ac.uk/PROSPERO/, Identifier CRD420250652843.

## 1 Introduction

The concept of sarcopenia was first introduced by Rosenberg in 1989 (1). As our understanding of the pathophysiology of sarcopenia deepened, the European Working Group on Sarcopenia in Older People formally defined sarcopenia in 2010 as a progressive geriatric syndrome characterized by age-related loss of muscle mass, decline in muscle strength, and/or deterioration in physical performance (2).

However, cancer-related muscle loss represents a distinct pathophysiological entity from age-related sarcopenia. While age-related sarcopenia primarily results from gradual neuromuscular and hormonal changes, cancer-related muscle wasting is driven by a complex interplay of tumor-derived factors (such as pro-inflammatory cytokines), metabolic alterations (like increased protein catabolism, insulin resistance), and treatment-related toxicities (3, 4). This distinction has critical implications for intervention design, as cancer-related sarcopenia may require more aggressive, multifaceted approaches to counteract the accelerated catabolic state. The high metabolic activity of rapidly growing tumor cells and the prolonged inflammatory response that results in cachexia can significantly impair protein synthesis and breakdown in cancer patients. This cancer-related muscle wasting is characterized by a hypercatabolic and hypoanabolic state, accelerating the decline of muscle mass and function beyond typical aging patterns (5). Factors such as anorexia, reduced physical activity, surgical stress, chemotherapy, and radiotherapy contribute significantly to its pathogenesis (6). The overall incidence of sarcopenia among cancer patients varies greatly (35.3, 28.3%–61%) (7) due to the lack of international agreement on sarcopenia assessment and diagnostic criteria thresholds. Extensive research data indicate that sarcopenia has significant prognostic value in cancer patients, including increased risk of postoperative complications, prolonged hospital stays, intolerance to anticancer therapies, reduced quality of life, and even decreased overall survival (8).

While radical tumor resection can alleviate cachexia in some advanced cancer patients, its impact on reversing established sarcopenia is often limited due to persistent metabolic dysregulation. Studies have shown that tumor-related factors are significantly associated with low grip strength, slowed gait speed, and reduced physical performance in patients (9). Additionally, tumor-induced loss of muscle mass may affect approximately 80% of advanced cancer patients and accounts for 30%–50% of tumor-related mortality factors (10). Therefore, early identification and intervention for sarcopenia in cancer patients are of paramount importance. Although researchers and medical professionals have gradually become more aware of the development of sarcopenia in cancer patients in recent years, the current healthcare delivery system is not set up to address the need for cancer-related sarcopenia prevention and control. Current sarcopenia guidelines recommend non-pharmacological interventions like exercise and nutritional support (11, 12). Given the distinct pathophysiology, the efficacy of these sarcopenia interventions specifically in the cancer population required dedicated synthesis. This research attempts to provide a systematic review and meta-analysis of the impact of sarcopenia interventions on muscle health in adult cancer survivors.

## 2 Methods

### 2.1 Registration

Our paper is registered on PROSPERO (CRD420250652843) on February 16th, 2025.

### 2.2 Data sources, search strategy, and definitions

We conducted a literature search in PubMed (Medline), Web of Science, Embase, Cochrane, Scopus, and EBSCOhost (CINAHL and PsycINFO) databases from 2010 (when sarcopenia was formally defined) to November 2, 2025. Search terms were sarcopenia interventions, adults, cancer, and muscle health indicators, encompassing muscle mass, muscle strength, and physical performance. We expanded the search terms related to sarcopenia interventions to include primarily exercise and nutrition. We focused on sarcopenia outcome measures such as muscle mass, muscle strength, and physical performance to ensure comprehensive retrieval. The search strategy was developed in consultation with a medical librarian to ensure comprehensiveness and minimize bias. The complete search strategies for all databases are provided in Table 1.

**Table 1:** Search strategies.

Databases	Strategies and results
Medline	#1 “Sarcopenia”[Mesh] OR sarcopeni* OR “muscular atrophy”[Mesh] OR “muscular atroph*” OR “muscle loss” OR “muscle wasting” OR “myopenia” OR “low muscle mass” OR “low skeletal muscle mass” OR “muscle depletion”—58790 #2 “Muscle Strength”[Mesh] OR “muscle strength” OR “hand strength”[Mesh] OR “grip strength” OR “physical performance” OR “walk* speed” OR “gait speed” OR “chair stand” OR “muscle function” OR “muscle quality”—127317 #3 “Resistance Training”[Mesh] OR “resistance train*” OR “strength train*” OR “weight train*” OR “exercise”[Mesh] OR “exercise therap*”[Mesh] OR exercise* OR “physical activit*”—741475 #4 “Dietary Proteins”[Mesh] OR “protein supplement*” OR “amino acids”[Mesh] OR “leucine” OR “HMB” OR “beta-hydroxy-beta-methylbutyrate” OR “nutritional support”[Mesh] OR “nutrition therapy” OR “dietary supplements”[Mesh]—1210336 #5 #3 OR #4—1930649 #6 “Neoplasms”[Mesh] OR neoplasm* OR cancer* OR tumor* OR tumour* OR oncolog* OR malignant*—6334487 #7 (#1 OR #2) AND #5 AND #6—4641 #8 (#1 OR #2) AND #5 AND #6 Filters: Full text, Randomized Controlled Trial, Humans, Adult: 19 + years, Exclude preprints, from 2010–2025—518
Web of Science	1: TS = (sarcopeni* OR “muscular atroph*” OR “muscle loss” OR “muscle wasting” OR myopenia OR “low muscle mass” OR “low skeletal muscle mass” OR “muscle depletion”)—58412 2: TS = (“muscle strength” OR “grip strength” OR “physical performance” OR “walk* speed” OR “gait speed” OR “chair stand” OR “muscle function” OR “muscle quality”)—121314 3: TS = (“resistance train*” OR “strength train*” OR “weight train*” OR exercise* OR “physical activity”)—842383 4: TS = (“protein supplement*” OR “dietary protein*” OR “amino acids” OR leucine OR HMB OR “beta-hydroxy-beta-methylbutyrate” OR “nutritional support” OR “nutrition therapy” OR “dietary supplement*”)—564540 5: #3 OR #4—1396490 6: TS = (neoplasm* OR cancer* OR tumor* OR tumour* OR oncolog* OR malignant*)—5168451 7: TS = (randomized OR randomised OR randomization OR randomisation OR placebo OR “clinical trial” OR randomly OR “random allocation” OR “controlled trial”)—2040128 8: (#1 OR #2) AND #5 AND #6 AND #7—1344 9: (((#1 OR #2) AND #5 AND #6 AND #7) NOT WC = (Pediatrics)) AND DT = (Article)—1011 10: (((#1 OR #2) AND #5 AND #6 AND #7) NOT WC = (Pediatrics)) AND DT = (Article) and 2010–2015 (Publication Years)—931
Embase	#1. ‘sarcopenia’/exp. OR ‘muscle atrophy’/exp. OR ‘muscle atrophy’:ti,ab OR ‘sarcopenia’:ti,ab OR ‘muscle loss’:ti,ab OR ‘muscle wasting’:ti,ab OR ‘myopenia’:ti,ab OR ‘low muscle mass’:ti,ab OR ‘low skeletal muscle mass’:ti,ab OR ‘muscle depletion’:ti,ab OR ‘muscle strength’/exp. OR ‘hand strength’/exp. OR ‘muscle strength’:ti,ab OR ‘grip strength’:ti,ab OR ‘physical performance’:ti,ab OR ‘walking speed’:ti,ab OR ‘gait speed’:ti,ab OR ‘chair stand’:ti,ab OR ‘muscle function’:ti,ab OR ‘muscle quality’:ti,ab—3593 #2. ‘resistance training’/exp. OR ‘resistance train*’:ti,ab OR ‘strength train*’:ti,ab OR ‘weight train*’:ti,ab OR ‘exercise therap*’:ti,ab OR ‘exercise*’:ti,ab OR ‘physical activit*’:ti,ab OR ‘physical activity’/exp. OR ‘diet protein’/exp. OR ‘protein supplement*’:ti,ab OR ‘dietary protein*’:ti,ab OR ‘amino acid’/exp. OR ‘leucine’:ti,ab OR ‘hmb’:ti,ab OR ‘beta-hydroxy-beta-methylbutyrate’:ti,ab OR ‘nutritional support’/exp. OR ‘nutrition therapy’:ti,ab OR ‘dietary supplement*’:ti,ab—3646840 #3. ‘neoplasm’/exp. OR ‘neoplasm*’:ti,ab OR ‘cancer*’:ti,ab OR ‘tumor*’:ti,ab OR ‘tumour*’:ti,ab OR ‘oncolog*’:ti,ab OR ‘malignant*’:ti,ab—8086396 #4. #1 AND #2 AND #3—10745 #5. #4 AND (‘human’/de OR ‘randomized controlled trial’/de) AND ([adult]/lim OR [aged]/lim OR [middle aged]/lim OR [very elderly]/lim OR [young adult]/lim) AND ‘Article’/it—3593 #6 #5 AND (2010:py OR 2011:py OR 2012:py OR 2013:py OR 2014:py OR 2015:py OR 2016:py OR 2017:py OR 2018:py OR 2019:py OR 2020:py OR 2021:py OR 2022:py OR 2023:py OR 2024:py OR 2025:py)—3263
CINAHL with full text	(sarcopeni* OR “muscular atroph*” OR “muscle loss” OR “muscle wasting” OR myopenia OR “low muscle mass” OR “low skeletal muscle mass” OR “muscle depletion” OR “muscle strength” OR “grip strength” OR “physical performance” OR “walk* speed” OR “gait speed” OR “chair stand” OR “muscle function” OR “muscle quality”) AND (“resistance train*” OR “strength train*” OR “weight train*” OR exercise* OR “physical activity” OR “protein supplement*” OR “dietary protein*” OR “amino acids” OR leucine OR HMB OR “beta-hydroxy-beta-methylbutyrate” OR “nutritional support” OR “nutrition therapy” OR “dietary supplement*”) AND (neoplasm* OR cancer* OR tumor* OR tumour* OR oncolog* OR malignant*) AND (randomized OR randomised OR randomization OR randomisation OR placebo OR “clinical trial” OR randomly OR “random allocation” OR “controlled trial”)—211
APA PsycInfo	(sarcopeni* OR “muscular atroph*” OR “muscle loss” OR “muscle wasting” OR myopenia OR “low muscle mass” OR “low skeletal muscle mass” OR “muscle depletion” OR “muscle strength” OR “grip strength” OR “physical performance” OR “walk* speed” OR “gait speed” OR “chair stand” OR “muscle function” OR “muscle quality”) AND (“resistance train*” OR “strength train*” OR “weight train*” OR exercise* OR “physical activity” OR “protein supplement*” OR “dietary protein*” OR “amino acids” OR leucine OR HMB OR “beta-hydroxy-beta-methylbutyrate” OR “nutritional support” OR “nutrition therapy” OR “dietary supplement*”) AND (neoplasm* OR cancer* OR tumor* OR tumour* OR oncolog* OR malignant*) AND (randomized OR randomised OR randomization OR randomisation OR placebo OR “clinical trial” OR randomly OR “random allocation” OR “controlled trial”)—69
Cochrane Central Registry of Controlled Trials	#1 MeSH descriptor: [Sarcopenia] explode all trees—1089 #2 MeSH descriptor: [Muscular Atrophy] explode all trees—1612 #3 (sarcopenia OR “muscular atroph” OR “muscle loss” OR “muscle wasting” OR myopenia OR “low muscle mass” OR “low skeletal muscle mass” OR “muscle depletion”):ti,ab,kw—4146 #4 (“muscle strength” OR “grip strength” OR “physical performance” OR “walk speed” OR “walking speed” OR “gait speed” OR “chair stand” OR “muscle function” OR “muscle quality”):ti,ab,kw—44699 #5 MeSH descriptor: [Muscle Strength] explode all trees—9820 #6 MeSH descriptor: [Hand Strength] explode all trees—2695 #7 MeSH descriptor: [Physical Functional Performance] explode all trees—691 #8 #1 OR #2 OR #3 OR #4 OR #5 OR #6 OR #7—48085 #9 (“resistance train” OR “strength train” OR “weight train” OR exercise OR “physical activity”):ti,ab,kw—173741 #10 MeSH descriptor: [Exercise] explode all trees—41320 #11 MeSH descriptor: [Resistance Training] explode all trees—6264 #12 (“protein supplement” OR “dietary protein” OR “amino acids” OR leucine OR HMB OR “beta-hydroxy-beta-methylbutyrate” OR “nutritional support” OR “nutrition therapy” OR “dietary supplement”):ti,ab,kw—16868 #13 MeSH descriptor: [Nutritional Support] explode all trees—4467 #14 #10 OR #11 OR #12 OR #13—192901 #15 (neoplasm OR cancer OR tumor OR tumour OR oncology OR malignant OR maglinancy):ti,ab,kw—273964 #16 MeSH descriptor: [Neoplasms] explode all trees—130416 #17 #15 OR #16—300492 #18 #8 AND #14 AND #17 trials—360
Scopus	(TITLE-ABS-KEY (sarcopeni* OR “muscular atroph*” OR “muscle loss” OR “muscle wasting” OR myopenia OR “low muscle mass” OR “low skeletal muscle mass” OR “muscle depletion” OR “muscle strength” OR “grip strength” OR “physical performance” OR “walk* speed” OR “gait speed” OR “chair stand” OR “muscle function” OR “muscle quality”) AND TITLE-ABS-KEY (“resistance train*” OR “strength train*” OR “weight train*” OR exercise* OR “physical activity” OR “protein supplement*” OR “dietary protein*” OR “amino acids” OR leucine OR HMB OR “beta-hydroxy-beta-methylbutyrate” OR “nutritional support” OR “nutrition therapy” OR “dietary supplement*”) AND TITLE-ABS-KEY (neoplasm* OR cancer* OR tumor* OR tumour* OR oncolog* OR malignant*) AND TITLE-ABS-KEY (randomized OR randomised OR randomization OR randomisation OR placebo OR “clinical trial” OR randomly OR “random allocation” OR “controlled trial”)) AND PUBYEAR > 2009 AND PUBYEAR < 2027 AND (LIMIT-TO (DOCTYPE, “ar”)) AND (LIMIT-TO (SRCTYPE, “j”))—2004

### 2.3 Study selection: inclusion and exclusion criteria

The PICOS framework was used to define the study eligibility criteria, as detailed below: ① P (Population): Adults (≥18 years) diagnosed with any cancer type, regardless of cancer stage, comorbidities, or metastasis. ② I (Intervention): Non-pharmacological interventions aimed at preventing or treating sarcopenia, as recommended by relevant guidelines (11, 12). These were categorized *post-hoc* for analysis as: Exercise-only interventions (e.g., resistance, aerobic, or mixed training). Nutrition-only interventions (e.g., protein, amino acids, caloric supplements). Multi-component interventions (combining exercise and nutritional supplementation, with or without additional components such as psychosocial or educational support). ③ C (Comparator): Usual care control groups. ④ O (Outcomes): At least one of the following muscle health indicators: muscle mass, muscle strength, or physical performance. ⑤ S (Study Design): Randomized Controlled Trials (RCTs). Since the term “sarcopenia” has been formally defined by EWGSOP since 2010, we restricted the time frame. The exclusion criteria were as follows: ① Studies that evaluated solely surgical, pharmaceutical, or hormonal interventions for sarcopenia. ② Studies where the intervention was exclusively educational or behavioral counseling without a structured exercise or nutritional supplementation component. ③ Abstracts, conference proceedings, reviews, meta-analyses, or non-peer-reviewed publications.

**Figure 1:**
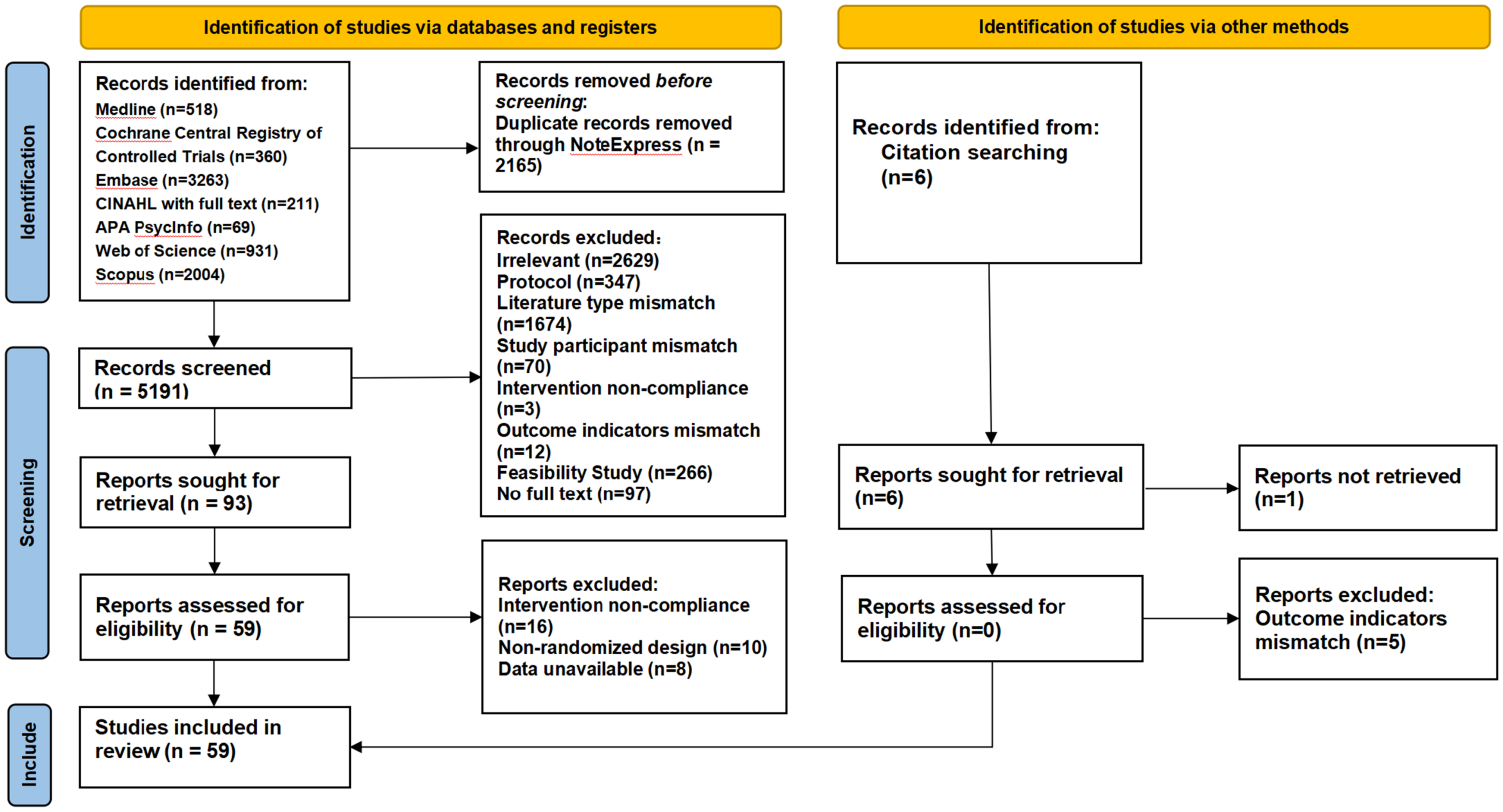
PRISMA flow diagram.

The study selection process was performed independently by two reviewers. First, they screened titles and abstracts against the inclusion criteria. Then, the full texts of potentially relevant studies were retrieved and assessed in detail. Any disagreements between the reviewers were resolved through discussion or by a third independent reviewer. This process is summarized in the PRISMA flow diagram (Figure 1).

### 2.4 Data extraction and organization

The extraction procedure was carried out separately by two reviewers. The extracted data included author and publication year, country, cancer type, sample size, study design, research setting, intervention components, muscle health measurement tools, and main conclusions. The association between sarcopenia interventions was measured using the standardized mean difference (SMD). The mean and SD changes in muscle mass, strength, and physical performance were extracted between baseline and post-intervention or follow-up assessments. For the primary analysis, we chose the model with the highest degree of control over confounders from each research. Cochrane guidelines were used when calculating the change in SD. A modest association is represented by a value of SMD = 0.2, a moderate connection by a value of SMD = 0.5, and a significant association by a value of SMD = 0.8.

### 2.5 Risk of bias assessment

To evaluate the studies’ methodological quality and risk of bias, we employed the Cochrane Risk of Bias Tool 2 Checklist. Two separate reviewers completed this assessment.

### 2.6 Statistical analysis

The meta and metafor packages in RStudio were used to analyze the data. Unless otherwise noted, a two-sided *p*-value of less than 0.05 was used to evaluate statistical significance. If a publication included subgroups (such as different intervention types and follow-up duration), we treated it as an independent study in the meta-analysis. The studies that were part of this meta-analysis were aggregated using SMD. *I*^2^ and *τ*^2^ statistics were used to assess the degree of heterogeneity among the studies. Based on the *I*^2^ value, a suitable effect summary model was selected after the heterogeneity analysis was finished. Significant heterogeneity was considered if the *I*^2^ value was ≥50% (or *p*-value <0.10), in which case the random-effects model was used. If the *I*^2^ value was <50%, we considered that the heterogeneity was acceptable, and the common-effects model was applied (13, 14). Due to the anticipated clinical and methodological heterogeneity, subgroup analyses were conducted. Sensitivity analyses (through leave-one-out analysis) and comprehensive publication bias assessments (Egger’s test, funnel plot, trim-and-fill method) were performed. For outcomes where Egger’s test indicated potential publication bias (*p* < 0.10), we applied the trim-and-fill method to estimate and adjust for the number of potentially missing studies and recalculated the pooled effect size. The results of these analyses are reported in the main text, and all funnel plots are visualized in Supplementary Figures 1–7.

## 3 Results

### 3.1 Characteristics of the included studies

Of the 7,356 articles identified from databases and reference searches, studies were omitted after removing 2,165 duplicates and excluding 2,629 irrelevant studies. The remaining 59 RCTs met the final inclusion criteria. Please refer to the PRISMA flow diagram (Figure 1) for detailed selection process.

Of the 59 studies included, 31 were from Europe (15–45), 12 from North America (46–57), 10 from Asia (58–67), and 6 from Australia (68–73). The majority of the included studies focused on breast cancer (19, 20, 22, 24, 40, 42, 46, 48–50, 53, 55, 58, 59, 67) (15 studies), followed by various cancer types (13 studies) (21, 23, 25, 27, 29, 32, 34, 36, 38, 45, 47, 54, 63), prostate cancer (10 studies) (35, 39, 44, 52, 64, 68–72), colorectal cancer (7 studies) (15–17, 26, 30, 61, 62), lung cancer (5 studies) (33, 41, 43, 60, 73), head and neck cancer (3 studies) (31, 37, 51), pancreatic (2 studies) (56, 66), and 1 study on bladder (18), gastric (28), gynecologic (65), and colon cancers (57). The interventions were predominantly multimodal, often combining exercise (aerobic, resistance, high-intensity interval training), nutritional support (dietary counseling, protein supplementation), and psychological or educational components. A summary of the characteristics of the studies is presented in Supplementary Table 1.

### 3.2 Significant improvement in mean muscle mass

**Figure 2:**
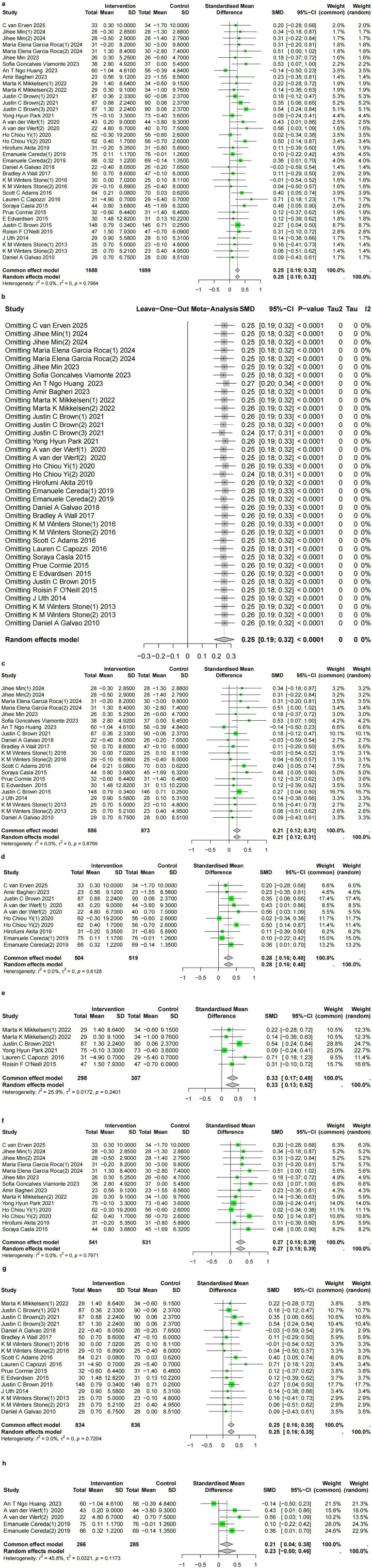
**(a)** Meta-analysis of sarcopenia interventions on muscle mass, using the common effects model. **(b)** Leave-one-out analysis of sarcopenia interventions on muscle mass. **(c)** Meta-analysis of sarcopenia intervention (exercise-only) on muscle mass. **(d)** Meta-analysis of sarcopenia intervention (nutrition-only) on muscle mass. **(e)** Meta-analysis of sarcopenia intervention (multi-components) on muscle mass. **(f)** Meta-analysis of sarcopenia intervention on muscle mass assessed by BIA. **(g)** Meta-analysis of sarcopenia intervention on muscle mass assessed by DXA. **(h)** Meta-analysis of sarcopenia intervention on muscle mass assessed by CT.

Pooled results based on 28 publications (15, 24, 27, 29, 30, 36, 39–41, 44, 48–53, 56, 59, 61, 62, 64–66, 68, 69, 71, 72) (37 studies, 3,387 participants) revealed a statistically significant preservation in muscle mass following sarcopenia interventions (SMD = 0.25; 95% CI, 0.18 to 0.32, $I_{\text{hetrogeneity}}^{2}$ = 0%, *p* = 0.8) (Figure 2a). The negligible heterogeneity suggests a consistent, positive effect of interventions on muscle mass across diverse cancer populations and intervention protocols. Sensitivity analysis results demonstrate robustness (Figure 2b). Egger’s test yielded a bias estimate of −0.33 (SE = 0.59), *p*-value = 0.58, indicating no publication bias. Visualization of the funnel plot for publication bias is presented in Supplementary Figure 1. Subgroup analyses indicated that multi-component interventions were associated with the greatest improvement (SMD = 0.33; 95% CI, 0.17 to 0.49, $I_{\text{hetrogeneity}}^{2}$ = 25.9%, *p* = 0.24) compared to exercise-only (SMD = 0.21; 95% CI, 0.12 to 0.31, $I_{\text{hetrogeneity}}^{2}$ = 0%, *p* = 0.88), or nutrition-only (SMD = 0.28; 95% CI, 0.16 to 0.4, $I_{\text{hetrogeneity}}^{2}$ = 0%, *p* = 0.61) interventions (Figures 2c–e). To address measurement heterogeneity, we conducted subgroup analyses by tool. When muscle mass was measured by BIA, the effect size of sarcopenia interventions on muscle mass improvement was significant (SMD = 0.27; 95% CI, 0.15 to 0.39, $I_{\text{hetrogeneity}}^{2}$ = 0%, *p* = 0.79). Similar effect sizes were observed when measured by DXA (SMD = 0.25; 95% CI, 0.16 to 0.35, $I_{\text{hetrogeneity}}^{2}$ = 0%, *p* = 0.72). The effect size was comparatively smallest when measured by CT (SMD = 0.21; 95% CI, 0.04 to 0.38, $I_{\text{hetrogeneity}}^{2}$ = 45.8%, *p* = 0.11).

### 3.3 Significant improvement in mean grip strength

**Figure 3:**
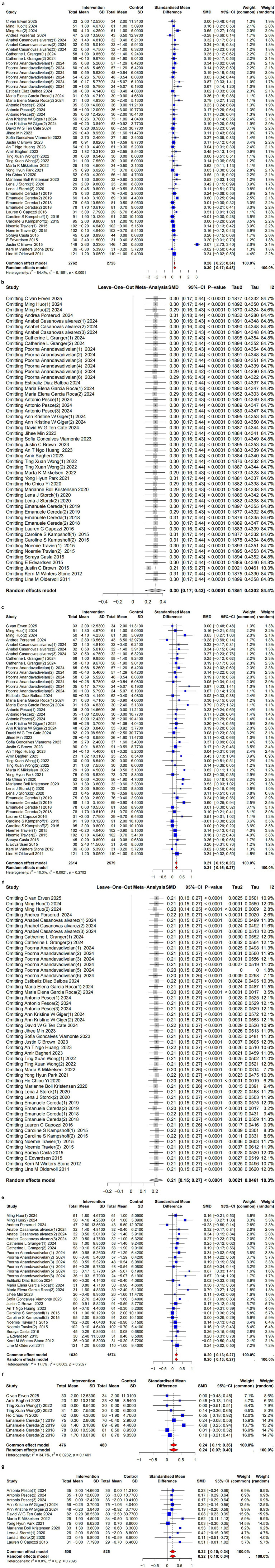
**(a)** Meta-analysis of sarcopenia interventions on grip strength, using the random effects model. **(b)** Leave-one-out analysis of sarcopenia interventions on grip strength. **(c)** Meta-analysis of sarcopenia interventions on grip strength after removing one study, using the common effects model. **(d)** Leave-one-out analysis of sarcopenia interventions on grip strength after removing one study. **(e)** Meta-analysis of sarcopenia interventions (exercise-only) on grip strength, using the common effects model. **(f)** Meta-analysis of sarcopenia interventions (nutrition-only) on grip strength, using the common effects model. **(g)** Meta-analysis of sarcopenia interventions (multi-components) on grip strength, using the common effects model.

Meta-analysis of 32 publications (15, 17–20, 22, 24–27, 29, 31, 32, 36–38, 40–42, 45, 46, 49, 51, 56–58, 61–65, 73) (51 studies, 5,193 participants) initially showed a statistically significant improvement in grip strength (SMD = 0.21; 95% CI, 0.15 to 0.26, $I_{\text{hetrogeneity}}^{2}$ = 10.3%, *p* = 0.27) (Figure 3c) after removing one study (49) contributing to high heterogeneity (sources of heterogeneity identified through leave-one-out analysis, Figure 3b). Sensitivity analysis results of the remaining studies are presented in Figure 3d, indicating robust findings. However, Egger’s test analysis indicated possible publication bias, with a bias estimate of 1.78 (SE = 0.61, *p*-value = 0.0054) (visualized in Supplementary Figure 2). Therefore, we performed trim-and-fill analysis, which imputed 12 theoretically missing studies. The adjusted pooled result remained statistically significant, though the effect size was attenuated (SMD = 0.13; 95% CI, 0.07 to 0.2; $I_{\text{hetrogeneity}}^{2}$ = 44.2%, *p* = 0.0001) (Supplementary Figure 3). This suggested that while the true effect size may be smaller than initially estimated, the conclusion that interventions improve grip strength is robust. Publication bias was not significant after trim-and-fill analysis (visualized in Supplementary Figure 4), with a bias estimate of 0.2 (SE = 0.74), *p*-value = 0.79. Subgroup analyses suggested that all three intervention types contributed to improvements, with nutrition-only (SMD = 0.24; 95% CI, 0.11 to 0.36, $I_{\text{hetrogeneity}}^{2}$ = 34.7%, *p* = 0.14) (Figure 3f) showing a similar estimate to multi-component (SMD = 0.22; 95% CI, 0.1 to 0.34, $I_{\text{hetrogeneity}}^{2}$ = 0%, *p* = 0.71) (Figure 3g) or exercise-only interventions (SMD = 0.2; 95% CI, 0.13 to 0.27, $I_{\text{hetrogeneity}}^{2}$ = 17.5%, *p* = 0.20) (Figure 3e).

### 3.4 Significant improvement in mean physical performance

**Figure 4:**
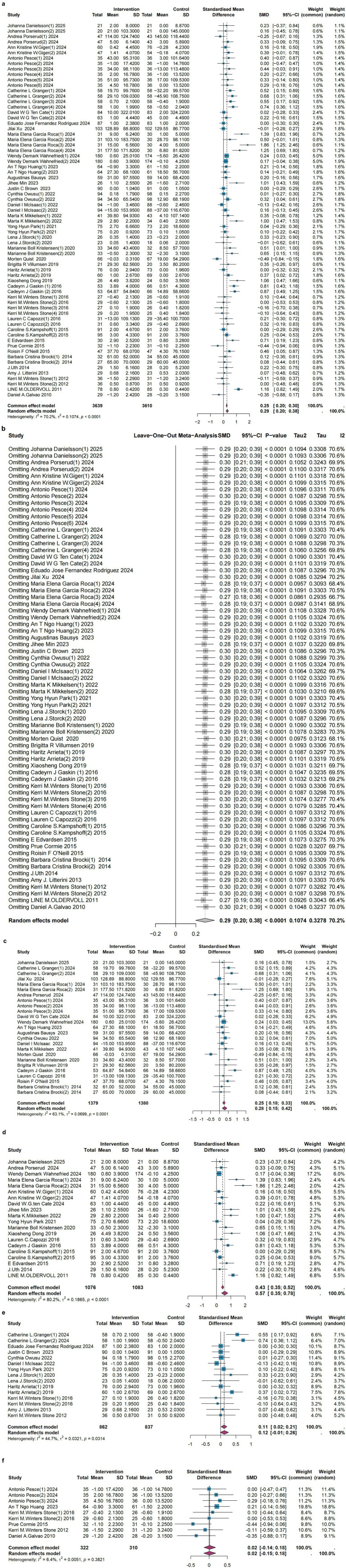
**(a)** Meta-analysis of sarcopenia interventions on physical performance, using the random effects model. **(b)** Leave-one-out analysis of sarcopenia interventions on physical performance. **(c)** Meta-analysis of sarcopenia interventions on 6-MWD, using the random effects model. **(d)** Meta-analysis of sarcopenia interventions on 30 s sit-to-stand test, using the random effects model. **(e)** Meta-analysis of sarcopenia interventions on SPPB scores, using the random effects model. **(f)** Meta-analysis of sarcopenia interventions on 5 times chair stand test, using the common effects model.

The meta-analysis results for sarcopenia interventions’ impact on physical performance [46 publications (16–18, 21, 23–26, 28, 29, 31–35, 38, 39, 41, 43–47, 51, 52, 54–57, 60, 61, 64, 67–70, 73), 69 studies, with 7,249 participants] indicate that sarcopenia interventions are beneficial for improving physical performance, despite significant heterogeneity (SMD = 0.29; 95% CI, 0.20 to 0.38; *I*^2^ = 70.2%; *p* < 0.0001) (Figure 4a). This high heterogeneity likely reflects the diversity of physical performance measures included and variations in patient populations and interventions. Sensitivity analysis confirmed the robustness of this finding (Figure 4b). Publication bias analysis yielded a bias estimate of 1.80 (SE = 0.77), *p*-value = 0.02, indicating possible publication bias (visualized in funnel plot Supplementary Figure 5). Therefore, we performed trim-and-fill analysis, which added 2 studies. The results remained significant (SMD = 0.26; 95% CI, 0.16 to 0.36; *I*^2^ = 74.3%; *p* < 0.0001) (Supplementary Figure 6). The Egger’s test result after trim-and-fill was: Bias estimate = 1.1 (SE = 0.83), *p*-value = 0.19 (visualized in funnel plot Supplementary Figure 7). Due to the diversity of measurement tools for physical performance, we conducted subgroup analyses for different measurement tools. Sarcopenia interventions were associated with 6-min walking distance (6-MWD) [20 publications (16–18, 23, 24, 26, 28, 29, 31, 33, 35, 39, 43, 51, 54–56, 60, 73), 25 studies, 2,759 participants; SMD = 0.28; 95% CI, 0.15 to 0.42, $I_{\text{hetrogeneity}}^{2}$ = 63.1%, *p* < 0.0001] (Figure 4c) and 30 s sit-to-stand test [17 publications (16, 18, 23–26, 29, 31, 38, 41, 44, 45, 51, 61, 64, 67, 70), 20 studies, 2,159 participants; SMD = 0.57; 95% CI, 0.35 to 0.78, *I*^2^ = 80.2%, *p* < 0.0001] (Figure 4d), though with significant heterogeneity. However, no statistically significant improvements were found for the short physical performance battery (SPPB) scores [11 publications (21, 32, 34, 46, 47, 52, 54, 55, 57, 64, 73), 15 studies, 1,699 participants, SMD = 0.12; 95% CI, −0.01 to 0.26, $I_{\text{hetrogeneity}}^{2}$ = 44.7%, *p* = 0.03) (Figure 4e] or the 5 times chair stand test (17, 46, 52, 56, 68, 69) (6 publications, 9 studies, 632 participants, SMD = 0.02; 95% CI, −0.15 to 0.18, $I_{\text{hetrogeneity}}^{2}$ =6.4%, *p* = 0.38) (Figure 4f).

### 3.5 Risk of bias, publication bias, and sensitivity analyses

The Cochrane Risk of Bias Tool 2 was used in this meta-analysis to evaluate the methodological quality of the included 59 publications (Supplementary Figure 8). In total, 17 studies had a low risk of bias (23, 28, 30, 34, 38, 39, 42, 46, 49, 50, 54, 55, 57, 63, 65, 70, 73), 36 had a potential risk of bias (15–21, 26, 27, 29, 31–33, 35–37, 40, 41, 43–45, 48, 51–53, 56, 58–61, 64, 66, 68, 69, 71, 72), and 6 had a high risk of bias (22, 24, 25, 47, 62, 67). The assignment allocation sequence was improperly hidden in 19 studies, and missing outcome data in 16 studies raised the possibility of bias. The lack of assessor blinding in 19 studies may have increased detection bias (Supplementary Figures 9a–d). As reported in sections 3.2–3.4, publication bias was assessed for each primary outcome, and where present, its impact was quantified and adjusted for using the trim-and-fill method. Sensitivity analyses via the leave-one-out method confirmed the robustness of the findings.

## 4 Discussion

This systematic review and meta-analysis of 59 RCTs demonstrates that non-pharmacological sarcopenia interventions are associated with statistically significant, though generally modest, improvements in muscle mass, grip strength, and certain measures of physical performance in adults with cancer. The effect sizes (SMDs ranging from 0.21 to 0.57 for significant outcomes) fall within the small-to-moderate range according to Cohen’s conventions. While these pooled effects may appear modest, they hold potential clinical importance. Even small improvements in muscle health can contribute to enhanced treatment tolerance, reduced incidence of dose-limiting toxicities, and better quality of life for cancer patients, a population particularly vulnerable to functional decline (8, 9).

Our subgroup analyses provide nuanced insights into intervention efficacy. The finding that multi-component interventions yielded the greatest improvement in muscle mass (SMD = 0.33) is biologically plausible and clinically significant. The synergistic effect of combining exercise (a potent anabolic stimulus) with nutritional support (providing substrate for muscle protein synthesis) directly counteracts the hypercatabolic and hypoanabolic state characteristic of cancer cachexia (4, 74). This supports the implementation of combined modality approaches as a more effective strategy for preserving muscle mass in this population. In terms of muscle strength, the similar effect sizes across intervention types (SMD = 0.20–0.24) suggest that different pathways may be involved. The effectiveness of nutrition-only interventions for grip strength was notable, particularly as many included studies involved breast cancer patients with upper extremity impairments post-surgery, for whom targeted exercise may be challenging. This highlights the role of adequate nutrition as a foundational component of sarcopenia management, even in the absence of structured exercise.

A critical finding of our analysis is the heterogeneity of effects across different physical performance measures. Interventions significantly improved the 6-MWD (SMD = 0.28) and the 30 s sit-to-stand test (SMD = 0.57), but not the SPPB or the 5-times chair stand test. The 6-MWD and 30 s sit-to-stand test are demanding, endurance or power-oriented tasks that may be more sensitive to change following exercise training. In contrast, the SPPB and 5-times chair stand test are brief, lower-intensity measures of basic functional mobility. The null findings for these latter tests may indicate that the interventions were not intense or specific enough to improve basic functional tasks, which might plateau early or be less responsive in cancer populations, especially those with advanced disease or high baseline function. Alternatively, these brief batteries may lack the sensitivity to detect meaningful change in this context. The substantial statistical heterogeneity observed across physical performance outcomes underscores the profound methodological variability in measuring this construct and calls for future consensus on optimal, cancer-sensitive functional endpoints.

When contextualizing our findings within the broader literature, it is important to distinguish our focus on cancer patients from previous reviews targeting age-related sarcopenia (75–77). The pathophysiology of muscle loss in cancer is more acute and driven by distinct inflammatory and metabolic factors. Our results align with reviews by Chang and Choo (78) and Kwon et al. (79), underscoring the value of combined interventions. However, they uniquely highlight the specific outcomes and challenges relevant to oncology populations, such as the impact of cancer type and treatment phase on intervention success.

### 4.1 Limitations and implications

This study has several limitations. First, we anticipated variation in how muscle mass, strength, and physical performance were defined and measured, as well as in the duration, frequency, intensity, and presence of supervision during the sarcopenia interventions. Although we extracted intervention components, the simplistic three-category classification (exercise, nutrition, multi-component) may not fully capture the complexity and heterogeneity of the interventions. Furthermore, not all of the aforementioned factors could be subjected to quantitative subgroup analyses due to inconsistent reporting. Even though the heterogeneity of partial results was minimal, the pooled data should be regarded cautiously due to the differences between studies. The generalizability of our findings may also be impacted by the diversity of our study population, which included patients receiving different treatments, at various stages of the disease, or in various settings. The potential effects of social and environmental circumstances on cancer patients’ muscle health warrant more consideration. Finally, while we employed rigorous methods to assess and adjust for publication bias, its potential impact, particularly for grip strength, cannot be fully discounted.

## 5 Conclusion

The prevalence of cancer-related loss of muscle mass or physical performance is predicted to rise in the upcoming years due to advancements in cancer treatment and an older population. Finding efficient ways to reduce the impact of cancer or its treatment on muscle loss is therefore essential. This meta-analysis provides evidence that sarcopenia interventions, especially multi-component programs, are associated with benefits for muscle health in adults with cancer. To encourage the evolution of reversible disease in a good way, policymakers and medical experts should concentrate on implementing sarcopenia interventions and dynamically evaluating muscle health markers. Future research should prioritize the development of standardized, cancer-specific core outcome sets for muscle health and should investigate the optimal dosing and timing of multi-component interventions within the cancer care continuum. These efforts may help this vulnerable group extend their progression-free survival and maintain functional independence.

## Data Availability

The original contributions presented in the study are included in the article/Supplementary material, further inquiries can be directed to the corresponding author.
