# Supplementary material for: Sarcopenia interventions targeted at improving muscle health in adults with cancer: a systematic review and meta-analysis
Source: Front Nutr. 2026 Jan 5;12:1671720. doi: 10.3389/fnut.2025.1671720 (PMC12812668; doi:10.3389/fnut.2025.1671720)
Supplement: Supplementary file 1 [file Supplementary_file_1.docx]

**Supplementary Materials**

eTable 1. Characteristics of Included Studies

| Source | Country | Cancer type | Participants, No. (Sex, No. [%]) | Age, mean (SD), y | Study design and settings | Intervention components (intervention group) | Tools for assessing muscle health indicators | Main conclusion |
| --- | --- | --- | --- | --- | --- | --- | --- | --- |
| C van Erven et al, 2025 | Netherlands | Colorectal | 67, 30 male [44%] | 70.5 (10) | RCT with newly diagnosed colorectal cancer adult patients enrolled from a teaching hospital in the Netherlands | Multimodal prehabilitation: 4-week personalized in-hospital supervised preoperative program including exercise, nutritional, psychological support, and a smoking cessation | Muscle mass: BIA  Muscle strength: handgrip | Positive impact of on preoperative nutritional status especially in body composition and muscle  strength |
| Johanna Danielsson et al, 2025 | Sweden | Colorectal | 52, 24 male [46%] | 77 (6) | RCT with older patients recruited from 4 hospitals in the Stockholm region: South General Hospital, Ersta Hospital, and Karolinska University Hospital in Solna and Huddinge | Home-based, high-intensity exercise, and partly supervised by a primary care physiotherapist consists of inspiratory muscle training, aerobic, and strength exercises for 2~3 weeks | Physical performance: 6MWD, 30-s chair stand test | Short-term exercise before colorectal cancer surgery can provide benefits in terms of increased postoperative inspiratory muscle strength, but could not on any other outcomes |
| Antonio Pesce et al, 2024 | Italy | Colorectal | 71, 42 male [59%] | 69 (9.2) | RCT with adult patients undergoing elective colorectal resection for colonic cancer enrolled from University Hospital of Ferrara | 3 supervised exercise sessions per week, protein intake 1.5 g/kg/day, psychological coping | Muscle strength: handgrip  Physical performance: 5-time sit to stand test, 6MWD | Feasible to implement a prehabilitation protocol lasting 4 weeks which may yield a signifcant improvement in the physical performance |
| Ming Huo et al, 2024 | China | Breast | 162, 0 male | 51.3 (11.2) | RCT with female patients with unilateral breast cancer enrolled from Beijing Chaoyang Sanhuan Cancer Hospital | Four shoulder patterns: flexion-abduction-external rotation; extension-adduction-internal rotation; flexion-adduction-external rotation; extension-abduction-internal rotation; | Muscle mass: supraspinatus muscle thickness  Muscle strength: handgrip | Positive clinical intervention effects in improving shoulder joint mobility disorders, pain, grip strength, and external rotation movement of the shoulder joint during chemotherapy after radical breast cancer surgery |
| Andrea Porserud et al, 2024 | Sweden | Urinary bladder | 90, 62 male [69%] | 71.5 (8.5) | RCT with adult patients scheduled for a robot-assisted radical cystectomy at Karolinska University Hospital, Sweden | Exercise with physiotherapist in primary care during the third week: aerobic and strengthening exercises, twice a week for 12 weeks, and daily walks | Muscle strength: handgrip  Physical performance: 30s sit-to-stand, 6MWD | Experienced additional positive effects on physical activity, fatigue, and health-related quality of life. |
| Anabel Casanovas-Álvarez et al, 2024 | Spain | Breast | 64, 0 male | 52 (11.8) | RCT with female patients aged 18~75 years receiving neoadjuvant therapy before surgery enrolled from a tertiary care hospital in Spain | Group-based prehabilitation  program consisting of Nordic walking, resistance training, and therapeutic education from month 4 of neoadjuvant therapy until before surgery. | Muscle strength: handgrip | Able to maintain arm function and increase functional capacity while decreasing fatigue in patients with breast cancer receiving neoadjuvant therapy |
| Catherine L Granger et al, 2024 | Australia | Lung | 116, 48 male [41.4%] | 66.4 (9.6) | RCT with adult patients enrolled from the lung cancer services of tertiary hospitals Royal Melbourne Hospital, St Vincent’s Hospital, and Austin Hospital in Melbourne, Australia | Postoperative 3-month  home-based exercise and self-management program, supported by weekly physiotherapist-led  telephone consultations | Muscle strength: handgrip  Physical performance: 6MWD | A postoperative home-based exercise and self-management program didn’t improve self-reported physical function in patients with lung cancer |
| Poorna Anandavadivelan et al, 2024 | Sweden | Breast | 206, 0 male | 53.3 (10.3) | RCT with female patients with unilateral breast cancer enrolled from Stockholm oncology clinics | trained twice a week on non-consecutive weekdays for 16 weeks, each session lasted approximately 60 min | Muscle strength: handgrip | Provides short-term benefits in reducing fatigue and maintaining physical function but limited long-term effects |
| Eduado Jose Fernandez‑Rodriguez et al 2024 | Spain | Various | 166, 85 male [51.2%] | 67 | RCT with patients enrolled from the Hospitalisation Unit of the Medical Oncology Service at the Complejo Hospitalario Universitario de Salamanca | Exercise re-education programme: Progressive mobilization; Gradation and Simplification of Activities of Daily Living: Teaching Techniques to Save Energy; Breathing exercises and incentive spirometry | Physical performance: SPPB | Beneficial effects of an exercise re-education programme, carried out by an interdisciplinary team in improving the autonomy of oncology patients with dyspnoea |
| Jihee Min et al, 2024 | Korea | Breast | 56, 0 male | 50.3 (6.6) | RCT with female patients enrolled from the Breast Cancer Center in Seoul, South Korea | Tailored, home-based stretching and resistance exercises using patients’ own body weight and 4 supervised exercise sessions at postoperative days 1-2, 7-10, 14-20, and 21-30, coinciding with surgeon visits | Muscle mass: BIA | 1-month tailored exercise program, initiated immediately after breast cancer surgery and supplemented with supervised sessions coinciding with surgeon visits, significantly improved shoulder function in patients with breast cancer |
| Estíbaliz Díaz-Balboa et al, 2024 | Spain | Breast | 122, 0 male | 48.9 (8.2) | RCT with female patients aged 18–70 years enrolled from cardiac rehabilitation and oncology units of the University Clinical Hospital of Santiago de Compostela (Galicia, Spain) | Physiotherapist-supervised exercise program in the cardiac rehabilitation ward (one-hour session, two days/  week, including strength training with body weight and elastic bands and aerobic exercise at 50–85% of heart rate reserve), during chemotherapy treatment | Muscle strength: handgrip | Cardio-Oncology Rehabilitation programs are safe and may help attenuate LVEF decline in BC women receiving cardiotoxic therapy and reduce BMI in obese patients |
| Wendy Demark-Wahnefried et al, 2024 | UK | Various | 381, 118 male [31%] | 69.8 (6.4) | RCT with older adult patients enrolled from the cancer survivors' homes across Alabama | One-year, home-based vegetable gardening intervention providing gardening supplies and mentorship by cooperative extension–certified master gardeners to plant and maintain spring, summer, and fall gardens | Physical performance: 2 minutes steps, 30s sit-to-stand | Didn’t significantly improve a composite index of diet, physical activity, and physical function but significantly increased vegetable and fruit consumption and experienced significant improvements in perceived health and physical performance |
| María Elena Garcia-Roca et al, 2024 | Spain | Breast | 59, 0 male | 49.5 (8.4) | RCT with female patients enrolled from the Hospital Provincial de Castellón | Online home-based exercise program consisted of a 60 min combined resistance and aerobic supervised exercise  session (6–8 points on Borg Scale CR-10, moderate intensity), twice a week during 24 weeks | Muscle mass: BIA  Muscle strength: handgrip  Physical performance: 6MWD, 30s sit-to-stand | An interesting strategy to improve physical fitness and adherence rates in breast cancer patients undergoing treatment |
| Ann-Kristine W Giger et al, 2024 | Denmark | Various | 182, 125 male [69%] | 77 (73-81) | RCT with older adult advance cancer patients enrolled from the oncologic outpatient clinic at Odense University Hospital | Comprehensive geriatric assessment and intervention: comorbidity, functional status, social support, medications, polypharmacy, cognition, psychosocial status, nutrition and weight loss, physical performance | Muscle strength: handgrip | Didn’t find improved physical performance or quality of life in older patients with frailty receiving CGA-guided interventions as an add-on to palliative-intent oncological treatment. |
| Jilai Xu et al, 2024 | China | Lung | 216 | 18~70 | RCT with postoperative adult patients enrolled from Beijing China-Japan Friendship Hospital and Cancer Hospital of Chinese Academy of Medical Science | Baduanjin qigong for 12 weeks | Physical performance: 6MWD | Baduanjin Qigong may have certain advantages in relieving cancer-related fatigue and FEV1%, and may be another new method of home-based pulmonary rehabilitation |
| Justin C Brown et al, 2024 | USA | Colon | 181, 95 male [52.5%] | 55.2 (12.8) | RCT with colon cancer adults enrolled from 3 US centers | Home-based weight-lifting exercises: 5 large muscle exercises (bench press, squats, 1-arm row, lunges, and deadlifts) performed in 3-5 sets of 6-10 repetitions at an intensity of 65%-85% of the 1-repetition maximum twice weekly for the duration of chemotherapy | Muscle strength: handgrip  Physical performance: SPPB | Didn’t prevent physical function decline during chemotherapy |
| David W G Ten Cate et al, 2024 | Netherlands | Colorectal | 251, 138 male [55%] | 70 (12.2) | RCT with adult patients undergoing elective colorectal resection for cancer enrolled from the Máxima Medical Center | 4-week out-patient setting supervised program containing four pillars. It consisted of three times a week supervised high intensity interval training, home based exercises on non-training days, consultation with a dietician  where dietary advices, protein and vitamin supplementation were provided, mental-health support and a preoperative smoking cessation program if applicable | Muscle strength: handgrip  Physical performance:  30s sit-to-stand, 6MWD | Multimodal prehabilitation is associated with improved postoperative functional capacity, even in patients without postoperative complications |
| Jihee Min et al, 2023 | Korea | Colorectal | 52, 23 male [44%] | 56.6 (8.9) | RCT with stage I-III colon or rectal cancer patients aged 19~70 years enrolled from the Colorectal Cancer Clinic, a tertiary referral center in Seoul, Korea | A 15-minute supervised exercise intervention twice a day under the guidance of a professor in the field of exercise oncology, three phases according to postoperative day and patients’ conditions | Muscle mass: BIA  Muscle strength: handgrip  Physical performance: 5-time sit to stand test | Postsurgical inpatient exercise may promote faster recovery and discharge after curative-intent colorectal cancer surgery |
| Sofia Gonçalves Viamonte et al, 2023 | Portugal | Various | 75, 17 male [22.7%] | 53.6 (12.3) | RCT with adult cancer survivors who had exposure to cardiotoxic cancer treatment and/or previous cardiovascular disease enrolled from a single hospital’s oncology and hematology departments | An individualized plan delivered by a nutritionist addressing dietary goals to improve control of modifiable cardiovascular risk factors (CVRFs);  A weekly group session (scheduled on the same days of the exercise sessions) on psychological management and lifestyle behavior change (addressing motivation for healthy lifestyle habits including regular physical activity);  A monthly health educational group session raising awareness of the importance of control of CVRFs; | Muscle mass: BIA  Muscle strength: handgrip  Physical performance: 60s sit-to-stand test | Greater improvements in peak VO2 compared with usual care encompassing an exercise intervention in a community setting, also showed superior results in exercise adherence, cardiovascular risk factor control, quality of life, and health literacy |
| Augustinas Bausys et al, 2023 | Lithuania | Gastric | 128, 77male [60%] | 62.5 (10.6) | RCT with gastric cancer adult patients enrolled from two centres in Lithuania | Exercise interventions focused on endurance, respiratory muscle strength, stretching, resistance training, nutritional and psychological support | Physical performance: 6MWD | Prehabilitation reduced morbidity in patients who underwent gastrectomy for gastric cancer |
| An T Ngo-Huang et al, 2023 | USA | Pancreatic | 151, 92 male [60.9%] | 66.2 (8.3) | RCT with adult patients enrolled from the University of Texas MD Anderson Cancer Center in Houston, Texas | Stretching, moderate intensity aerobic exercise, resistance exercises based on the American College of Sports Medicine (ACSM)  guidelines | Muscle mass: CT  Muscle strength: handgrip  Physical performance: 5-time sit to stand test, 6MWD | Highlighting the importance of activity among patients preparing for surgery |
| Amir Bagheri et al, 2023 | Iran | Colorectal | 46, 32 male [69.6%] | 58.4 (9.9) | RCT with adult patients enrolled from the Imam Khomeini hospital complex, Tehran, Iran | Mediterranean diet regime with extra virgin olive oil | Muscle mass: BIA  Muscle strength: handgrip | Mediterranean diet might be a strategy to improve nutritional status, quality of life, inflammatory markers, and body composition in patients with colorectal cancer cachexia |
| Cynthia Owusu et al, 2022 | USA | Breast | 213, 0 male | 71.9 (5.9) | RCT with older African American and non-Hispanic White stage I to III breast cancer survivors enrolled from the Gathering Place, a community cancer support center located in Beachwood | 20 weeks of supervised moderate-intensity aerobic and resistance training followed by 32 weeks of unsupervised exercise called IMPROVE | Physical performance: 6MWD, SPPB | Combined aerobic and resistance exercise appears to improve physical performance in older breast cancer survivors |
| Daniel I McIsaac et al, 2022 | Canada | Various | 182, 103 male [56.6%] | 74 (6.5) | RCT with older adult patients enrolled from the Ottawa Hospital, Ottawa, Ontario | A remotely supported, home-based exercise prehabilitation program, nutritional guidance | Physical performance: 6MWD, SPPB | Didn’t significantly improve postoperative recovery or other outcomes in older adults with frailty having cancer surgery. Program adherence may be a key mediator of prehabilitation efficacy |
| Ting Xuan Wong et al, 2022 | Malaysia | Breast and Colorectal | 91, 22 male [24.2%] | 60.3 (13.9) | RCT with patients aged 25~65 years enrolled from the surgical outpatient clinics of Hospital Tuanku Ja’afar, Negeri Sembilan and Hospital Kuala Lumpur | Standard milk-based formula containing macronutrients and fortified  with micronutrients | Muscle strength: handgrip | Modest benefits in attenuating weight loss whilst postoperative supplementation up to 90-days post-discharge improved handgrip strength and inflammatory prognostic markers |
| Marta K Mikkelsen et al, 2022 | Denmark | Various | 84, 36 male [42.9%] | 72 (68~75) | RCT with older advanced cancer patients enrolled from the Department of Oncology at Copenhagen University Hospital, Herlev and Gentofte Hospital | 12-week multimodal exercise-based program including supervised exercise twice weekly followed by a protein supplement, a home-based walking program, and nurse-led support and counseling | Muscle mass: DXA and BIA  Muscle strength: handgrip  Physical performance: 6MWD, 30s sit-to-stand test | Effective in improving physical function in older patients with advanced cancer during oncological treatment |
| Justin C Brown et al, 2021 | USA | Breast | 351, 0 male | 59.4 (8.7) | RCT with female patients enrolled from the Penn TREC Survivorship Center | Exercise treatment condition: in-person and home-based resistance and aerobic exercise;  Diet treatment condition: 24 weekly sessions of lifestyle modification instruction led by a registered dietitian | Muscle mass: DXA | Diet alone or diet plus exercise produced clinically meaningful weight loss at week 52 |
| Yong Hyun Park et al, 2021 | Korea | Prostate | 172, 172 male [100%] | 66.4 (7.5) | RCT with male patients enrolled from three hospitals | A smartphone application providing a personalized exercise program, daily activity monitoring, and diet counselling | Muscle mass: BIA  Muscle strength: handgrip  Physical performance: 2MWD, SPPB, 30s sit-to-stand | Effective method for the management of androgen deprivation therapy related adverse effects, with positive effects on cardiorespiratory endurance, sarcopenic obesity, and health-related quality of life. The use of Internet of Things-based technology can potentially  maximize the beneficial effect of lifestyle interventions |
| A van der Werf et al, 2020 | Netherlands | Colorectal | 107, 66 male [63%] | 65 (11) | RCT with patients scheduled for first-line chemotherapy enrolled from five Dutch hospitals | Individualized face-to-face and telephone consultations by a trained study dietitian. Face-to-face counselling was planned at every chemotherapy cycle during the study period, starting at the first cycle | Muscle mass: CT | Individualized nutritional counseling by a dietitian had no effect on muscle mass, but may increase body weight and improve progression free survival and overall survival |
| Marianne Boll Kristensen et al, 2020 | Denmark | Head and neck | 71, 46 male [64.8%] | 64.3 (8.2) | RCT with adult patients enrolled from the Danish Head and Neck Cancer Group’s national clinical quality database | Five-day initial residential stay and two-days follow-up residential stay after three months and consisted of group-based patient education sessions and a few individual activities | Muscle strength: handgrip  Physical performance: 6MWD, 30s sit-to-stand test | A multidisciplinary residential nutritional rehabilitation program had no effect on body weight in head and neck cancer survivors with self-reported interest in participation but may have effect on physical function and quality of life |
| Lena J Storck et al, 2020 | Switzerland | Various | 52, 29 male [55.8%] | 63.1 (10.3) | RCT with adult advanced patients enrolled from the cancer center at the Kantonsspital Winterthur | A 12-week leucine-rich supplement in combination with a nutrition and physical exercise program | Muscle strength: handgrip  Physical performance: SPPB, 60s sit-to-stand test | A good adherence to the multimodal therapy, significant improvement of handgrip strength in patients with advanced cancer, but no significant improvements in further physical performance tests, especially our primary outcome factor SPPB as well as nutritional status, fatigue or quality of life |
| Ho Chiou Yi et al, 2020 | Malaysia | Gynecologic | 118, 0 male | 50.3 (12) | RCT with adult cancer patients enrolled from the National Cancer Institute, Putrajaya | Preoperative whey protein-infused sole carbohydrate loading (CHO) and postoperative early oral feeding | Muscle mass: body composition analyzer  Muscle strength: handgrip | High compliance on the multimodal ERAS with preoperative whey protein-infused CHO loading and postoperative early oral feeding shortened the length of postoperative hospital stay without increasing complications |
| Morten Quist et al, 2020 | Denmark | Lung | 218, 107 male [49.1%] | 64.4 (8.5) | RCT with adult advanced cancer patients enrolled from the Department of Oncology, Rigshospitalet, University of Copenhagen | A 12-week supervised, structured exercise training program (aerobic, strength, and relaxation training) twice weekly | Physical performance: 6MWD | A significant reduction in the level of anxiety and depression and a significant increase in all muscle strength outcomes in the intervention group |
| Haritz Arrieta et al, 2019 | Spain | Various | 301, 121 male [39.4%] | 76.7 (5.0) | RCT with older patients aged more than 70 years enrolled from 12 recruiting centers in France | 1 year phoned physical activity advice individually adapted to physical assessment (twice a month during the first 6 months and thenmonthly) | Physical performance: SPPB | Personalized phoned physical activity advice had not reduced functional decline at 1 year but provided preliminary evidence that may prevent physical performance decline at 2 years |
| Brigitta R Villumsen et al, 2019 | Denmark | Prostate | 46, 46 male [100%] | 68.7 (4.6) | RCT with older patients enrolled from the urological outpatient clinics at Regional Hospital Holstebro and Regional Hospital Viborg | A 12 weeks of unsupervised homebased ‘exergaming’ (i.e., technology-driven exercise) | Physical performance: 6MWD | Unsupervised home-based ‘exergaming’ for 12 weeks had an effect on the primary outcome of 6MWT  ‘Exergaming’ intervention appeared safe and could be an alternative to traditional aerobic and resistance training |
| Hirofumi Akita et al, 2019 | Japan | Pancreatic | 62, 27 male [43.5%] | 67.1 (10.2) | RCT with adult patients aged 20~80 years enrolled from Osaka International Cancer Institute | Received 2 bottles/day (550 kcal/day) of an Eicosapentaenoic acid-enriched nutrition supplement during neoadjuvant chemoradiotherapy (NACRT) | Muscle mass: BIA | Could potentially improve the nutritional status of patients that received NACRT, but it was difficult for many patients to drink, due to its disagreeable taste |
| Emanuele Cereda et al, 2019 | Italy | Various | 166, 100 male [60.2%] | 65.4 (11.5) | RCT with malnourished advanced cancer patients enrolled from the Nutrition and Dietetics Service, Fondazione IRCCS Policlinico San Matteo, Pavia, Italy | Receive nutritional counseling with or without whey proteins isolate supplementation for 3 months | Muscle mass: CT  Muscle strength: handgrip | Improved body composition, muscle strength, body weight, and reduced chemotherapy toxicity |
| Xiaosheng Dong et al 2019 | China | Breast | 50, 0 male | 49.7 (6.7) | RCT with postoperative patients recruited from the Department of Breast Surgery, The Second Hospital of Shandong University, Jinan City, Shandong Province | Muscle training, Cardiorespiratory capacity training, Postoperative breast cancer rehabilitation knowledge | Physical performance: 30s sit-to-stand test | The combined exercise intervention based on internet and social media software offered rehabilitative effects in quality of life and muscle strength of postoperative patients with breast cancer in China |
| Daniel A Galvão et al, 2018 | Australia | Prostate | 57, 57 male [100%] | 70 (8.4) | RCT with bone metastases patients enrolled from Perth, Western Australia by local oncologists and urologists | Multimodal supervised aerobic, resistance, and flexibility exercises undertaken thrice weekly for 3 months | Muscle mass: DXA | Improvements in physical function and objectively measured lower body muscle strength with no skeletal complications or increased bone pain |
| Emanuele Cereda et al, 2018 | Italy | Head and neck | 159, 114 male [71.7%] | 65.1 (13.6) | RCT with patients enrolled from Nutrition and Dietetics Service, Fondazione IRCCS Policlinico San Matteo, Pavia | Nutritional counseling in combination with oral nutritional supplements (ONS) | Muscle strength: handgrip | Better weight maintenance, increased protein-calorie intake, improved quality of life and was associated with better anti-cancer treatment tolerance |
| Bradley A Wall et al, 2017 | Australia | Prostate | 97, 97 male [100%] | 69.1 (8.9) | RCT with male patients aged 43~90 years enrolled from Perth, Western Australia | Supervised exercise was undertaken twice weekly  at moderate to high intensity for 6 months | Muscle mass: DXA | A 6-month combined aerobic and resistance exercise program has a significant favorable effect on cardiorespiratory capacity, resting fat oxidation, glucose, and body composition despite the adverse effects of hormone suppression |
| Kerri M Winters-Stone et al, 2016 | USA | Prostate | 64, 64 male [100%] | 71.8 (7.2) | RCT with male patients recruited by the Oregon Health & Science University | A progressive strength training program: 1-h group exercise sessions twice weekly for 6 months | Muscle mass: DXA  Physical performance: 5-time sit to stand test, SPPB | Exercising Together is a novel couples-based  approach to exercise that was feasible and improved several health outcomes for both prostate cancer survivors and their spouses |
| Lauren C Capozzi et al, 2016 | Canada | Head and neck | 60, 49 male [81.7%] | 56.1 (9.2) | RCT with adult patients recruited through the head and neck clinic | A 12-week lifestyle intervention and progressive resistancetraining program either during radiation treatment or immediately after completion | Muscle mass: DXA  Muscle strength: handgrip  Physical performance: 6MWD, 30s sit-to-stand test | Didn’t reduce the loss of lean body mass, delaying the exercise program until after treatment completion was associated with improved intervention adherence, a finding with important clinical implications |
| Scott C Adams et al, 2016 | Canada | Breast | 200, 0 male | 48.8 (25–78) | RCT with female adult patients recruited from regional cancer centers in Edmonton, Ottawa, and Vancouver | Thrice-weekly supervised exercise sessions for the  duration of chemotherapy;  AET prescription: 60 mins (i.e., 15 mins at 60 % of peak oxygen uptake (VO2peak) and 45 mins at 80 % of VO2peak) of either  treadmill, cycle ergometer, or elliptical-based exercise;  RET prescription: 2 sets of 8–12 repetitions of 9 exercises, which were performed between 60 and 70 % of their predicted 1 repetition maximum and progressed throughout the intervention period | Muscle mass: DXA | RETspecific benefits in improving sarcopenia and dynapenia status in breast cancer patients initiating adjuvant chemotherapy |
| Cadeyrn J. Gaskin et al, 2016 | Australia | Prostate | 119 | 66.4 (8.2) | RCT with male adult recruited by local clinicians | A 12-week, clinician-referred, community-based exercise training program with supervised and unsupervised sessions | 6-MWD, 30s sit-to-stand test | Men with prostate cancer who act upon clinician referrals to community-based exercise training programs can improve their strength, physical functioning, and, potentially, cardiovascular health, irrespective of whether or not they are  treated with |
| Justin C Brown et al, 2015 | USA | Breast | 295, 0 male | 56 (8.8) | RCT with female adult patients enrolled by Philadelphia, Pennsylvnia, United States, 17033 Penn State Cancer Institute | A 12-month membership to a community fitness center， each session included stretching of major muscle  groups, cardiovascular warm-up, abdominal and lower back strengthening exercises, and weight lifting exercises | Muscle mass: DXA  Muscle strength: handgrip | Demonstrate the feasibility and potential efficacy for slowly progressive weight lifting to attenuate the decline of appendicular skeletal muscle mass among breast cancer survivors |
| Caroline S Kampshoff et al, 2015 | Netherlands | Various | 277, 55 male [19.9%] | 53.7 (11) | RCT with adult patients recruited from nine Dutch hospitals | A 12-week high intensity (HI) and low-to-moderate (LMI) intensity resistance and endurance exercise program | Muscle strength: handgrip | May be a dose–response relationship between exercise intensity and peakVO2, favoring HI exercise. HI and LMI exercise were equally effective in reducing general and physical fatigue |
| Roisin F. O’Neill et al, 2015 | UK | Prostate | 94 ,94 male [100%] | 69.8 (6.9) | RCT with adult male patients recruited from the Northern Ireland Cancer Centre | A dietary and exercise intervention, commensurate with UK healthy eating and physical activity recommendations for 6 months | Muscle mass: using the Durnin/Wormersley caliper method  Physical performance: 6MWD | Minimize the adverse body composition changes  associated with androgen deprivation therapy |
| Soraya Casla et al, 2015 | Spain | Breast | 89, 0 male | 49.1 (8.8) | RCT with adult female patients aged 29~69 years developed by collaboration between different institutions | Exercise program: twice-weekly supervised exercise sessions combining aerobic and resistance exercise that was increased in intensity over 12 weeks in a familiar and trusted environment, which promoted socialization between participants  Nutrition program: three workshops where specific concepts of nutrition and diet were explained | Muscle mass: BIA  Muscle strength: handgrip | A combined aerobic and  resistance exercise intervention results in statistically and clinically significant improvement in VO2max in breast cancer survivors, as well as improvements in muscle strength, body composition, quality of life, and fatigue |
| Prue Cormie et al, 2015 | Australia | Prostate | 63, 63 male [100%] | 68.4 (7.1) | RCT with adult male patients aged 46~80 years referred by oncologists and urologists in Perth, Western Australia | A 3-month supervised exercise programme involving aerobic and resistance exercise sessions commenced within 10 days of their first androgen-deprivation therapy injection | Muscle mass: DXA  Physical performance: 30s sit-to-stand test | Commencing a supervised exercise programme involving aerobic and resistance exercise when initiating androgen-deprivation therapy significantly reduced treatment toxicity, while improving social functioning and mental health |
| E Edvardsen et al, 2015 | Norway | Lung | 61, 28 male [45.9%] | 65.2 (8.9) | RCT with adult patients aged 18~80 years conducted at Oslo University Hospital in Norway | High-intensity endurance and strength training (60 min, three times a week, 20 weeks), starting 5~7 weeks after surgery | Muscle mass: DXA  Muscle strength: handgrip Physical performance: 30s sit-to-stand test | High-intensity endurance and strength training was well tolerated and induced clinically significant improvements in peak oxygen uptake, carbon monoxide transfer factor, muscular strength, total muscle mass, functional fitness and quality of life |
| Noémie Travier et al, 2015 | Netherlands | Breast | 204, 0 male | 49.6 (8) | RCT with female patients aged 25~75 years conducted in seven hospitals | An 18-week, two aerobic and strength exercise sessions per week, supervised by aphysiotherapist and incorporating cognitive behavioral principles of social Bandura’s cognitive theory | Muscle strength: handgrip | Positive effects on physical fatigue, submaximal cardiorespiratory fitness, and muscle strength |
| Barbara Cristina Brocki et al, 2014 | Denmark | Lung | 78, 46 male [59%] | 64.5 (9.5) | RCT with adult patients conducted at Aalborg University Hospital | Supervised out-patient exercise training sessions, one hour once a week for ten weeks. The sessions were based on aerobic exercises with target intensity of 60–80% of work capacity, resistance training and dyspnoea management | Physical performance: 6MWD | Supervised compared to unsupervised exercise training resulted in no improvement in healthrelated quality of life, except for the bodily pain domain, four months after lung cancer surgery. No effects of the intervention were found for any outcome after one year |
| J. Uth et al, 2014 | Denmark | Prostate | 57, 57 male [100%] | 66.8 (6.1) | RCT with adult male advanced cancer patients aged 18~76 presenting at Copenhagen Prostate Cancer Center, Copenhagen University Hospital, Rigshospitalet, or the Department of Urology, Frederiksberg Hospital | A 12 weeks two to three times weekly on a natural grass pitch:  the first 4 week: two weekly sessions, started with 15 min of warm-up exercises (running, dribbling, passing, shooting, balance, and muscle strength exercises) followed by 2×15 min of 5~7 a-side small-sided games.  In weeks 5~8: the duration of each session increased to 3×15-min games after the warm-up  In weeks 9~12: three weekly  training sessions of the same duration | Muscle mass: DXA  Physical performance: 30s sit-to-stand test | Football training over 12 weeks improved lean body mass and muscle strength compared with usual care in men with prostate cancer receiving androgen deprivation therapy |
| K.M. Winters-Stone et al, 2013 | USA | Breast | 71, 0 male | 46.4 (4.9) | RCT with prematurely menopausal female patients conducted by the Oregon Health & Science University | Impact+resistance training (prevent osteoporosis with impact+resistance) for 1 year | Muscle mass: DXA | Impact+resistance training may effectively combat bone loss and worsening body composition from premature menopause |
| Amy J. Litteriniet al, 2013 | USA | Advanced | 66, 30 male [45.5%] | 62.4 (12.5) | RCT with adult patients recruited to attend an oncology-specific exercise program at a hospital-based fitness facility between February 2010 and March 2012 | Exercise at a rate of 10 to 12  (or fairly light) on the Borg Rating of Perceived Exertion Scale, oxygen saturation levels were monitored | Physical performance: SPPB | Individuals with advanced cancer appear to benefit from exercise for improving functional mobility |
| Kerri M. Winters-Stone et al, 2012 | USA | Breast | 106, 0 male | 62.2 (6.7) | RCT with older postmenopausal female patients conducted by the Oregon Health & Science University | An exercise program consisting of two 1-hr supervised classes and one 1-hr home-based session per week for 1 year | Muscle strength: handgrip  Physical performance: SPPB, 5-time sit-to-stand test | Superior to stretching at improving maximal muscle strength and exercise adherence contributes to the degree of improvement |
| LINE M. OLDERVOLL et al, 2011 | Norway | Various | 231, 87 male [37.7%] | 62.4 (11) | RCT with advanced cancer patients enrolled from 6 sites | Physical exercise group exercised under supervision  60 minutes twice a week for 8 weeks | Muscle strength: handgrip  Physical performance: 30s sit-to-stand test | Physical performance was clinically and statistically significantly improved after 8 weeks of physical exercise |
| Daniel A et al, 2010 | Australia | Prostate | 57, 57 male [100%] | 69.8 (7.2) | RCT with male patients enrolled from Sir Charles Gairdner Hospital | Combined progressive resistance and aerobic training twice a week for 12 weeks | Muscle mass: DXA  Physical performance: 5-time sit-to-stand test | A relatively brief exposure to exercise significantly improved muscle mass, strength, physical function, and balance |

BIA: bio impedance analysis; 6MWD: 6-Minute Walking Distance; 2MWD: 2-Minute Walking Distance; SPPB: short physical performance battery; CT: computed tomography; DXA: Dual-emission X-ray Absorptiometry; ERAS: Enhanced Recovery after Surgery; RET: resistance exercise training; AET: aerobic exercise training

**eFigure 1: Funnel plots for visual inspection of Publication Bias**


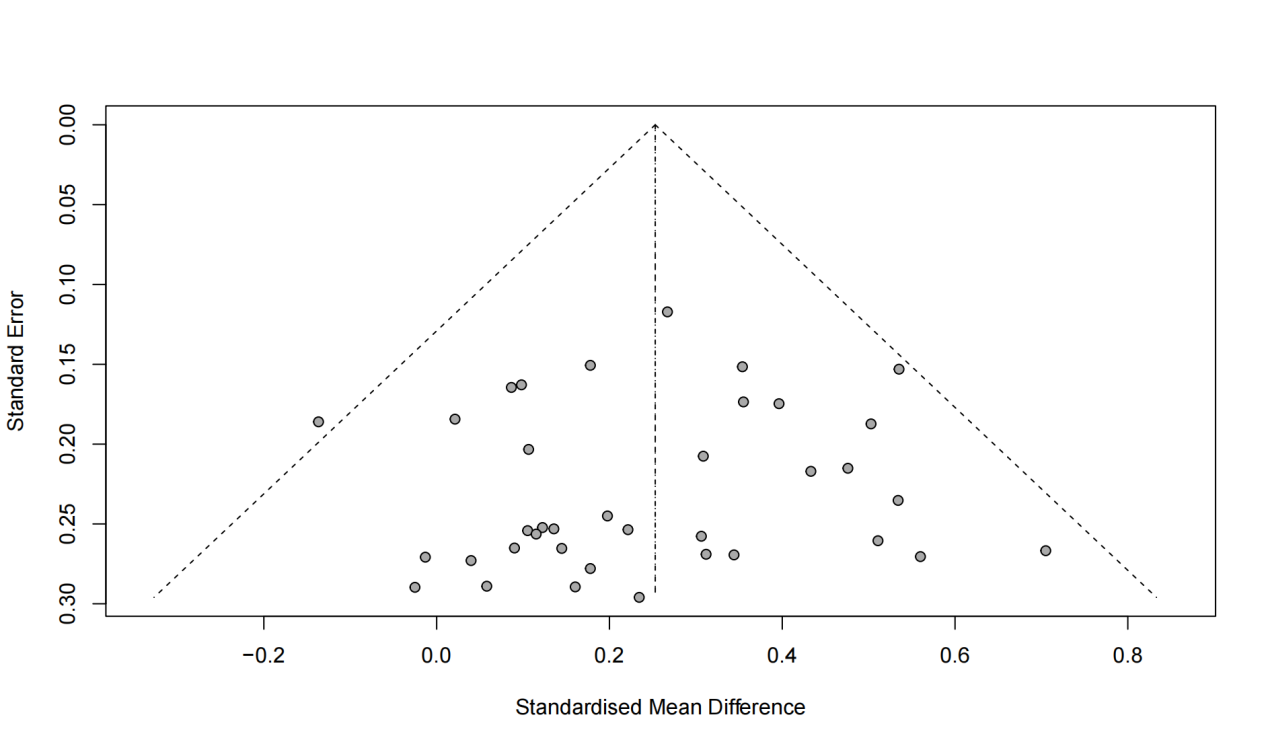


eFigure 1 Publication Bias in Studies assessing Sarcopenia Interventions’ effect on Muscle Mass


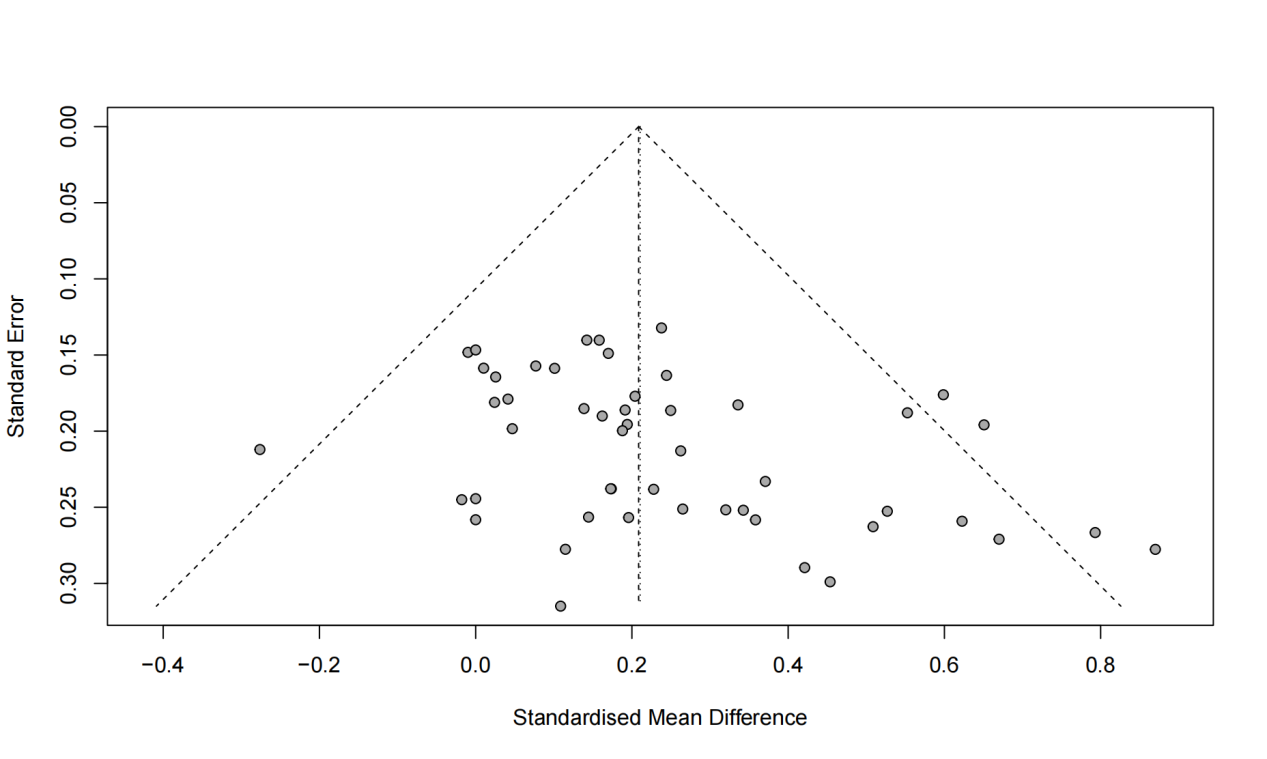


eFigure 2 Publication Bias in Studies assessing Sarcopenia Interventions’ effect on Grip Strength


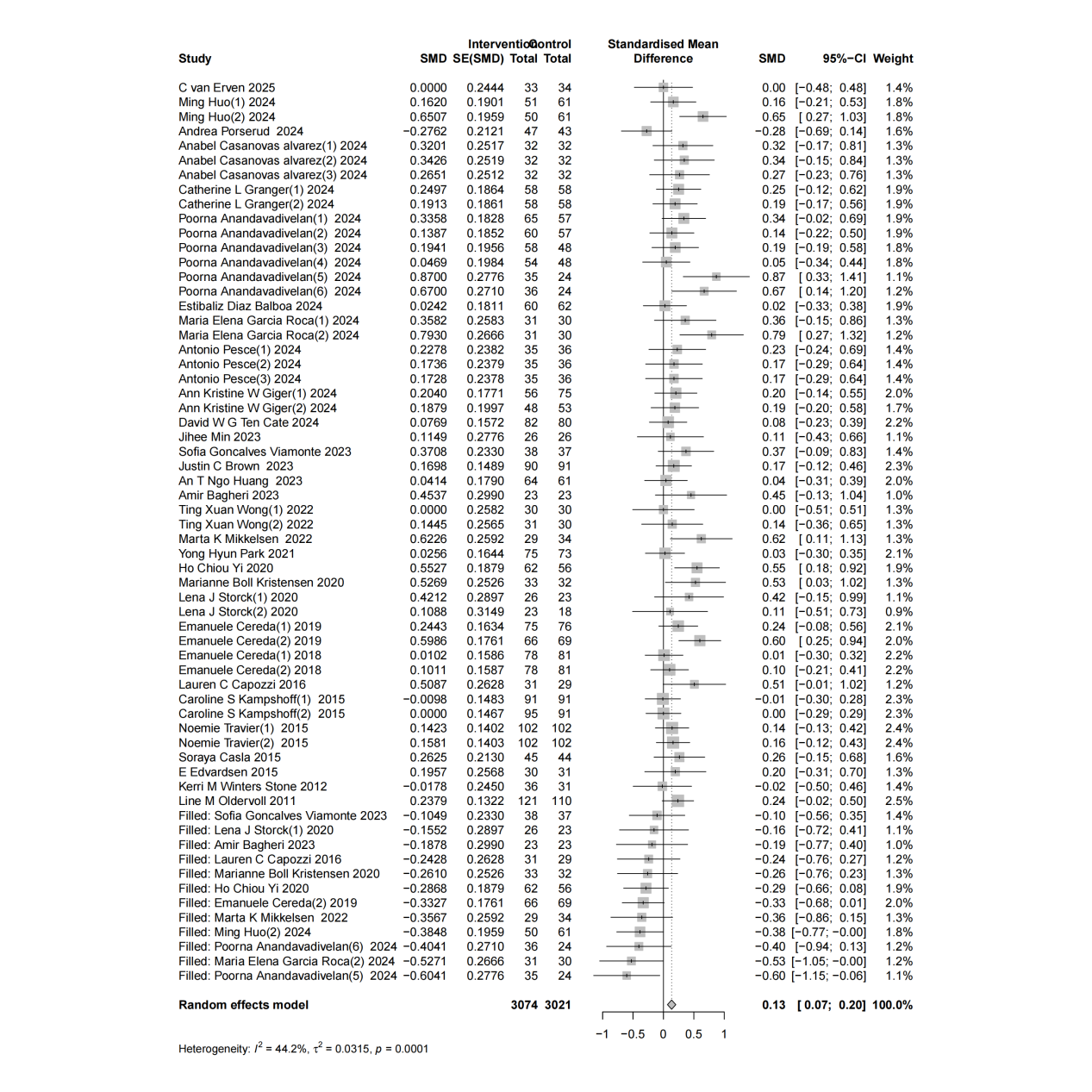


eFigure 3 Trim-and-fill analysis for Studies assessing Sarcopenia Interventions’ effect on Grip Strength


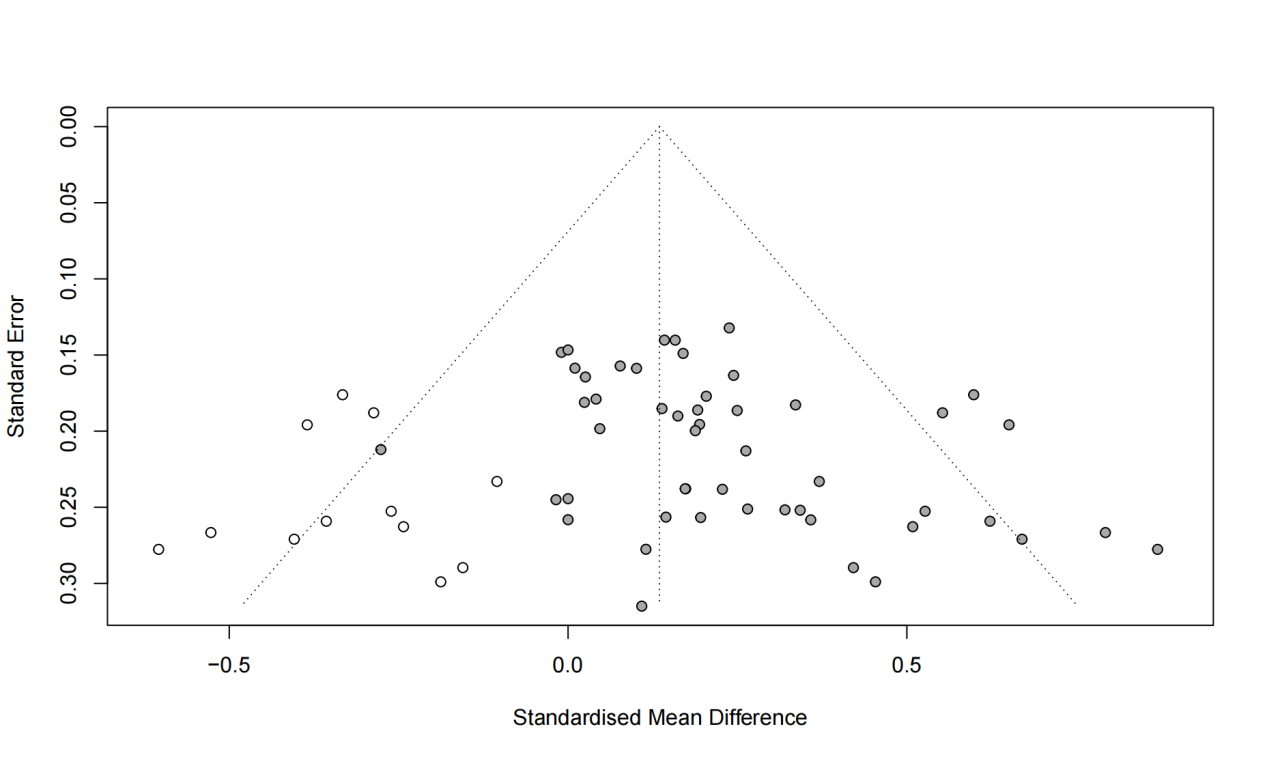


eFigure 4 Trim-and-fill analysis for Publication Bias in Studies assessing Sarcopenia Interventions’ effect on Grip Strength


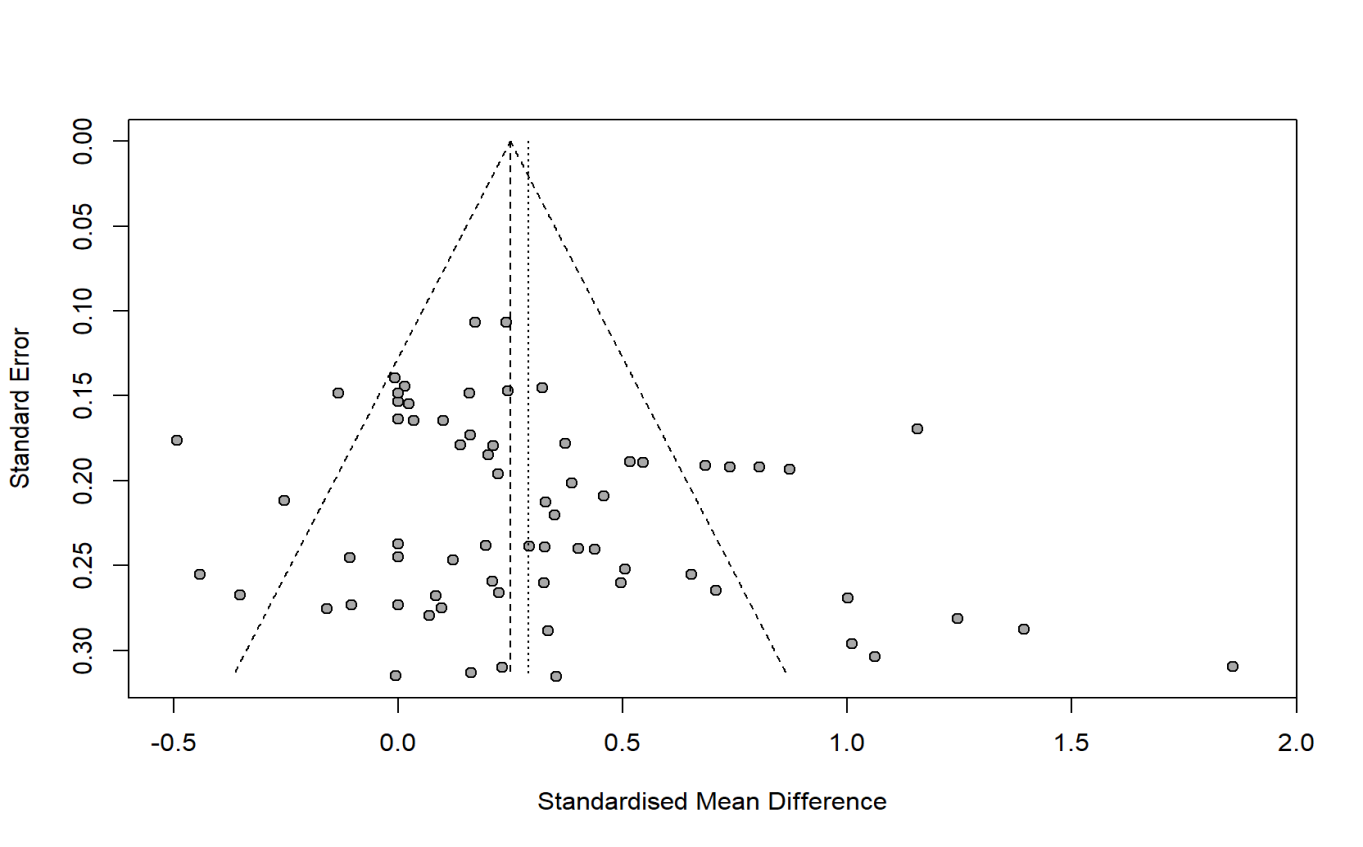


eFigure 5 Publication Bias in Studies assessing Sarcopenia Interventions’ effect on Physical Performance


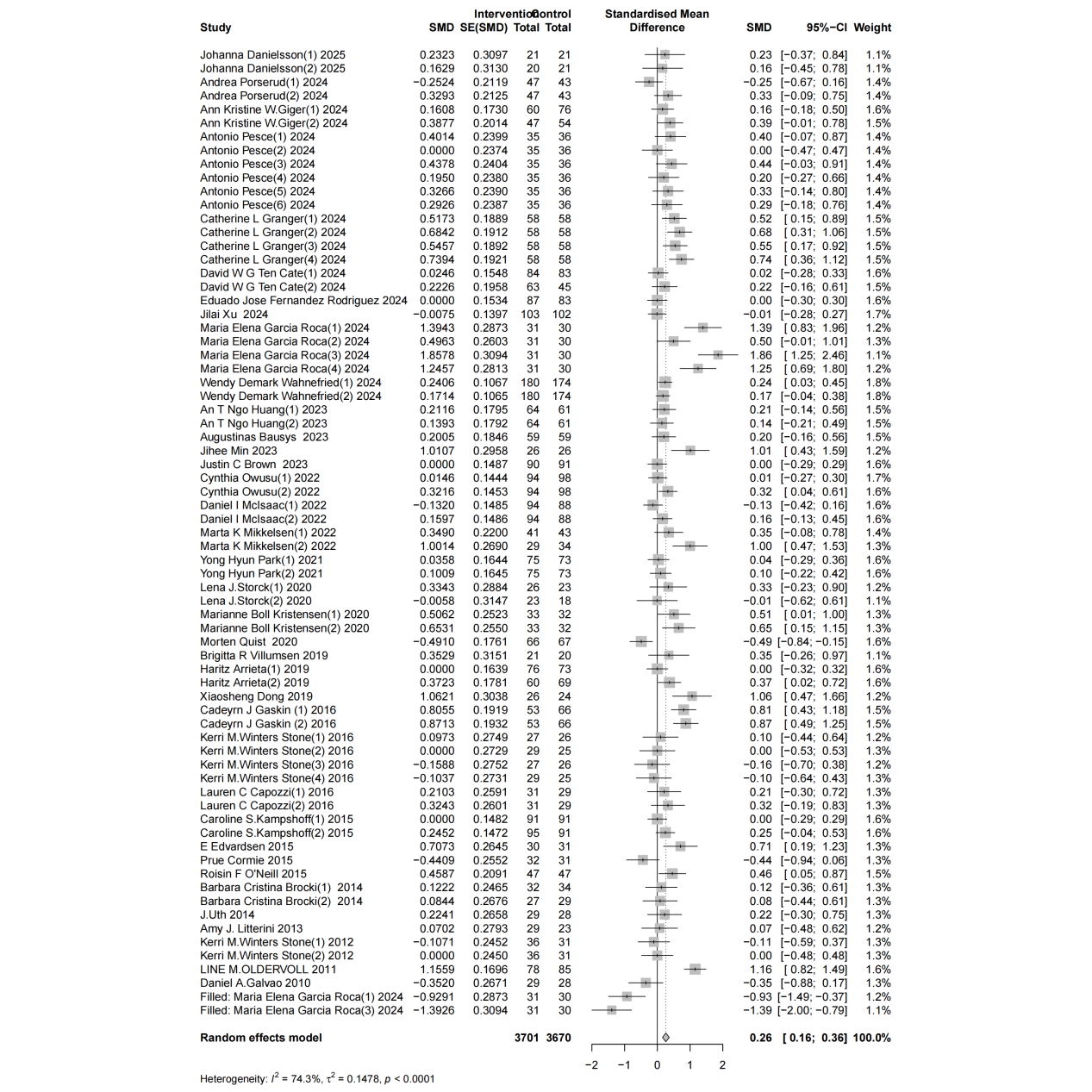


eFigure 6 Trim-and-fill analysis for Studies assessing Sarcopenia Interventions’ effect on Physical Performance


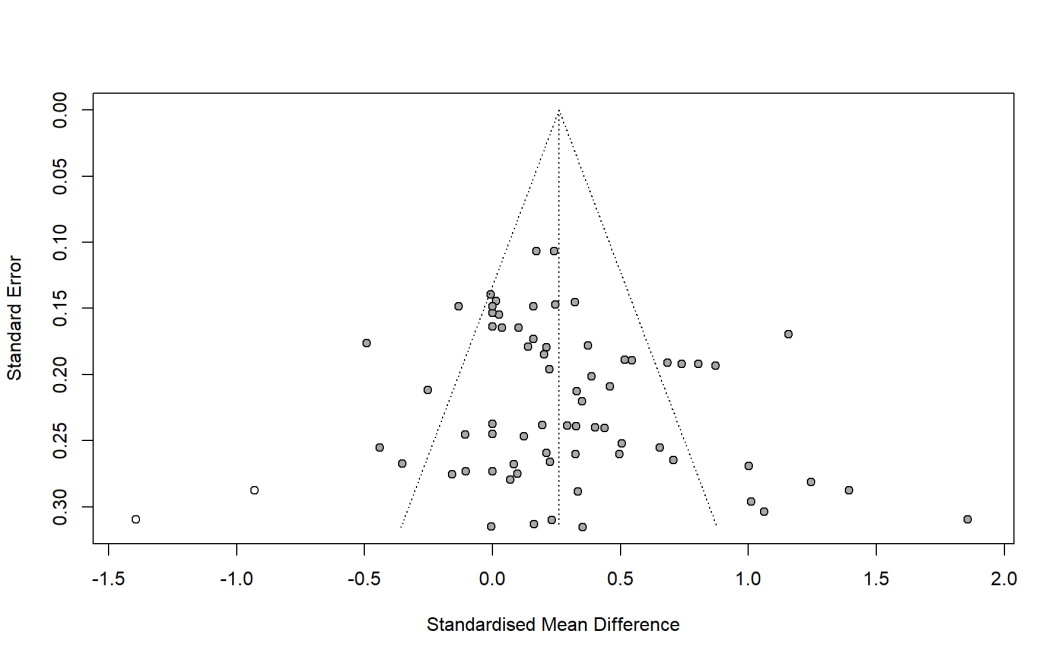


eFigure 7 Trim-and-fill analysis for Publication Bias in Studies assessing Sarcopenia Interventions’ effect on Physical Performance

**eFigure 8: Quality assessment of included studies using the Cochrane risk-of-bias tool 2**


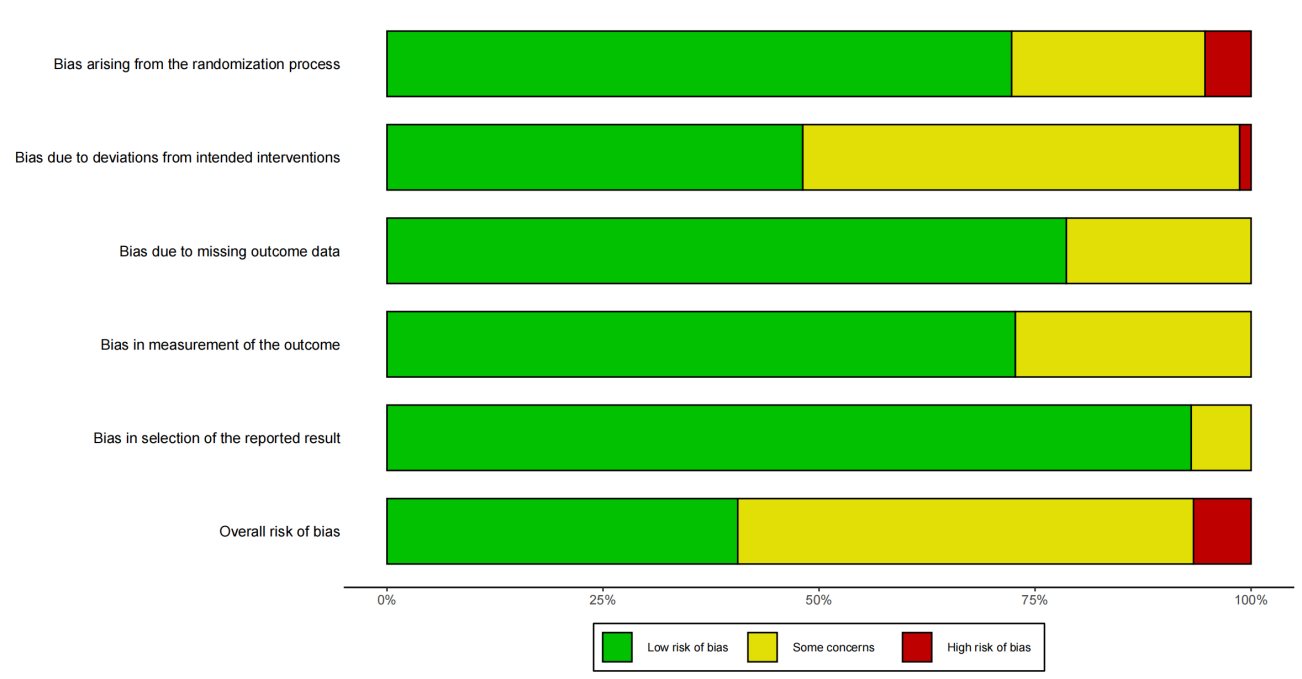


eFigure 8 Risk of Bias


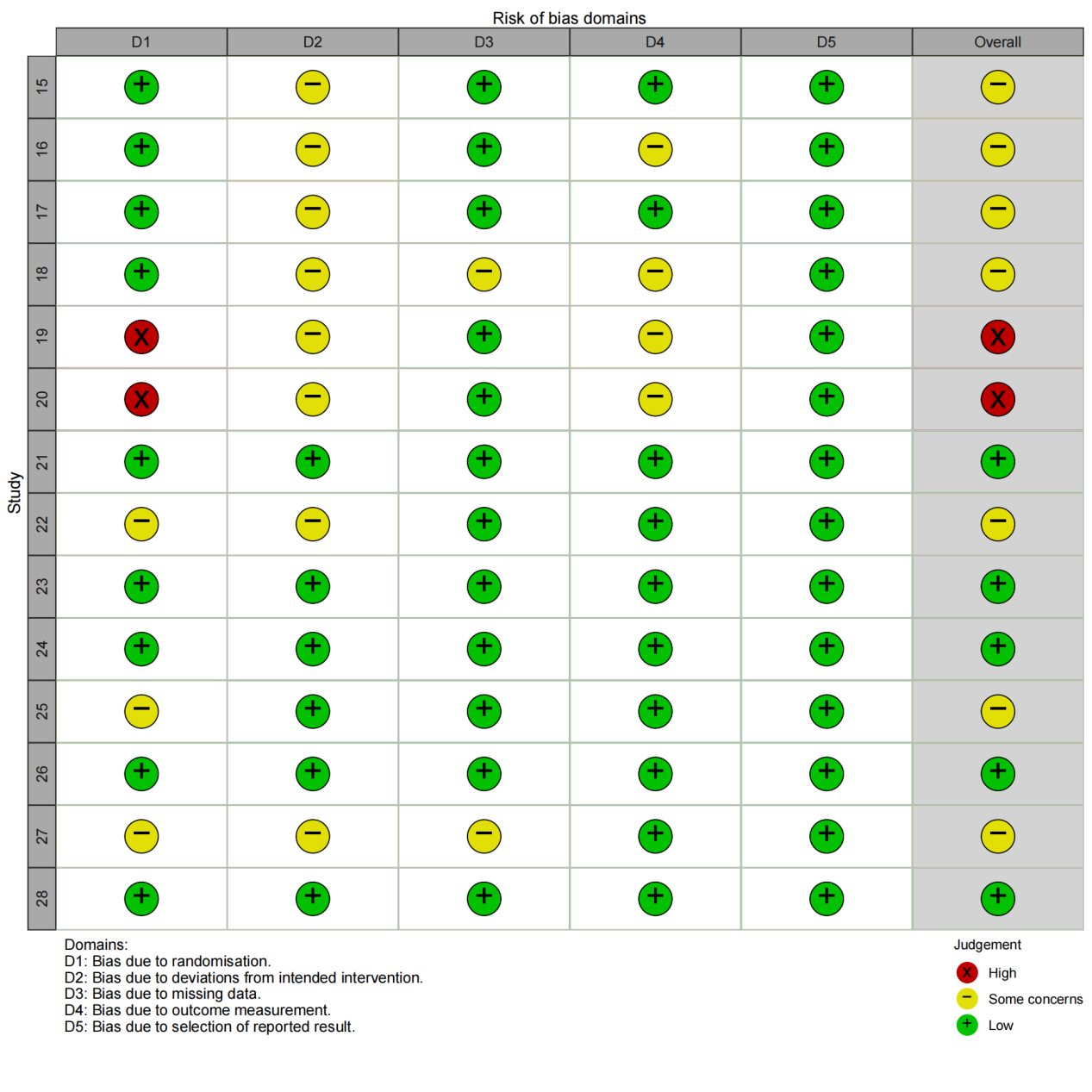


eFigure 9a Traffic lights summary of References 15~28


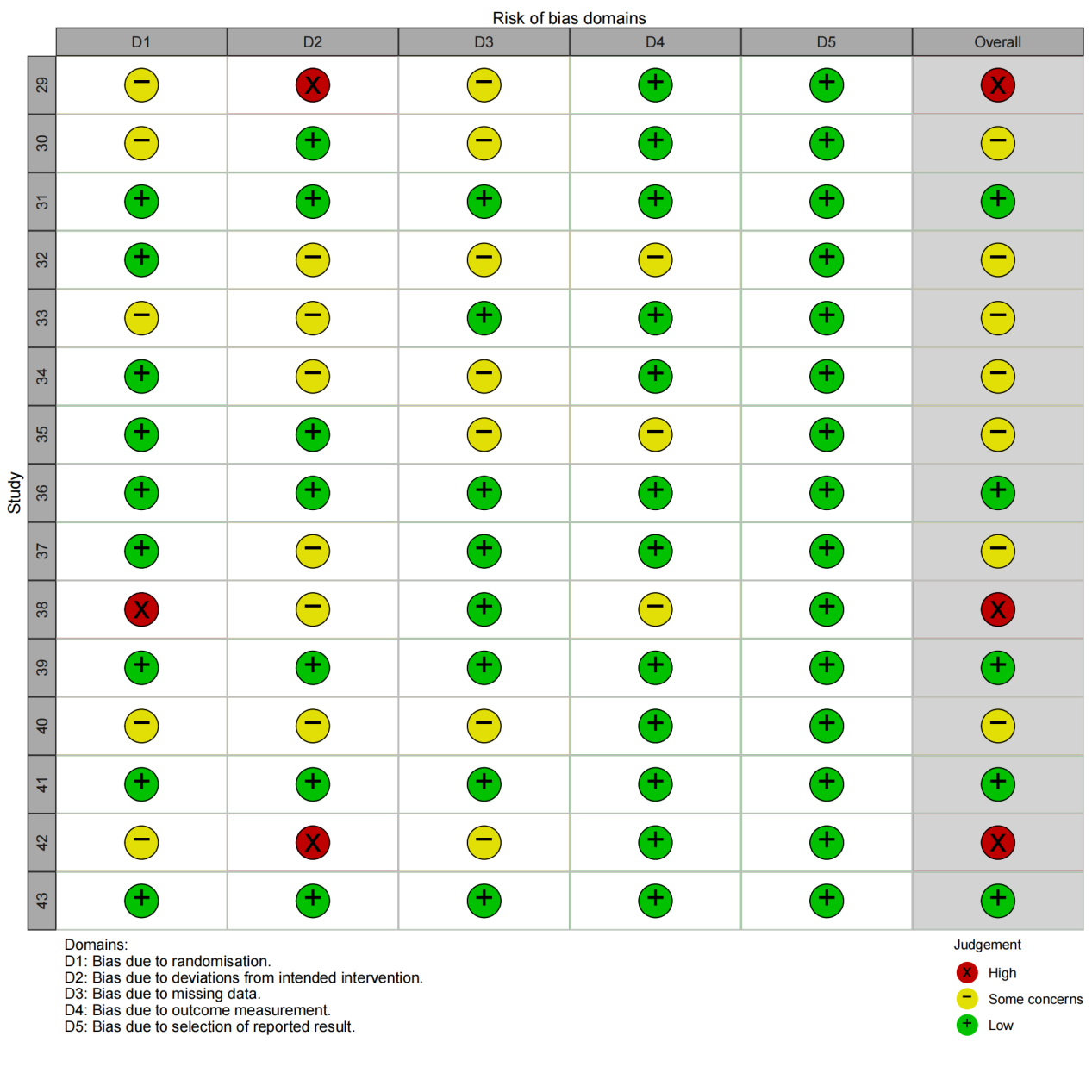


eFigure 9b Traffic lights summary of References 29~43


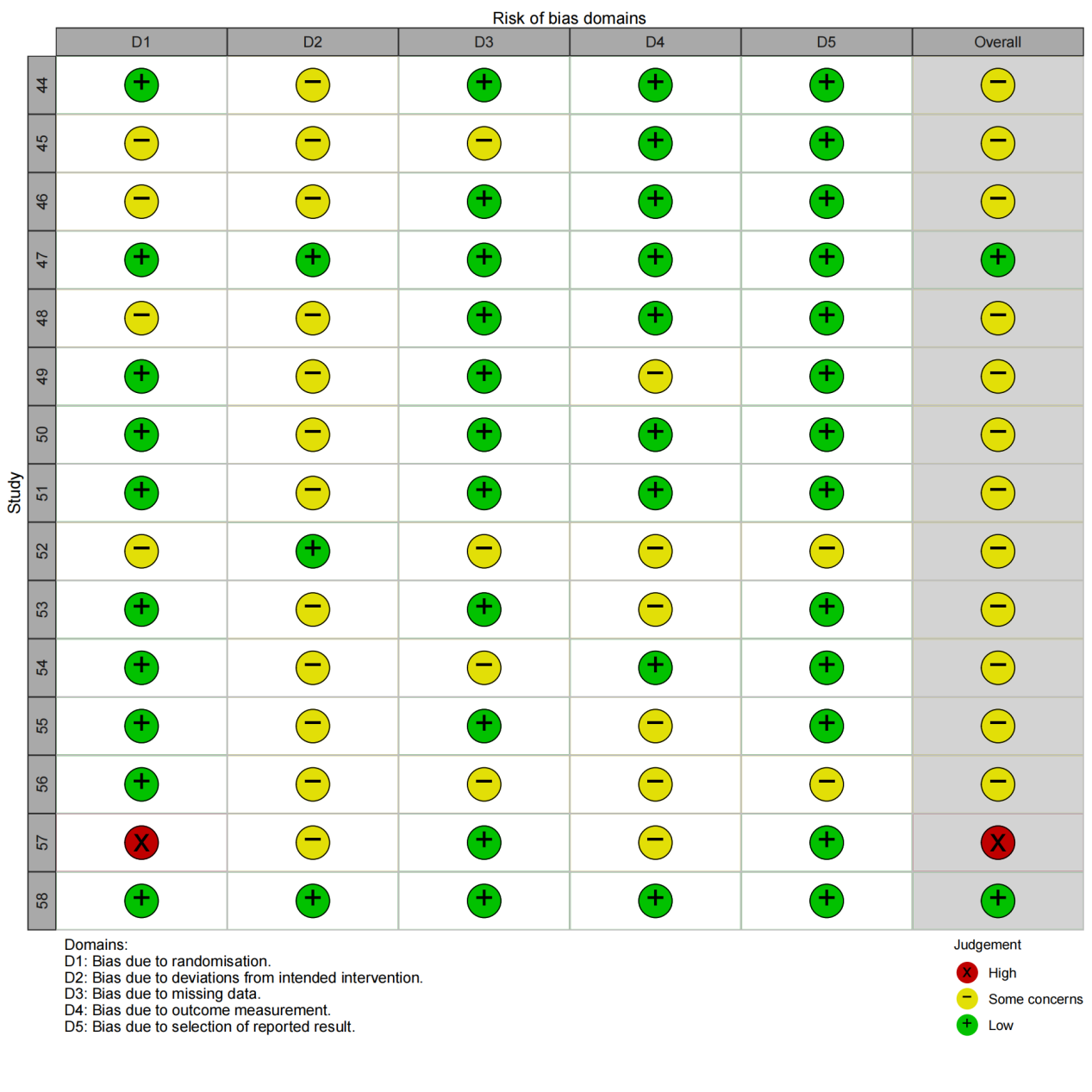


eFigure 9c Traffic lights summary of References 44~58


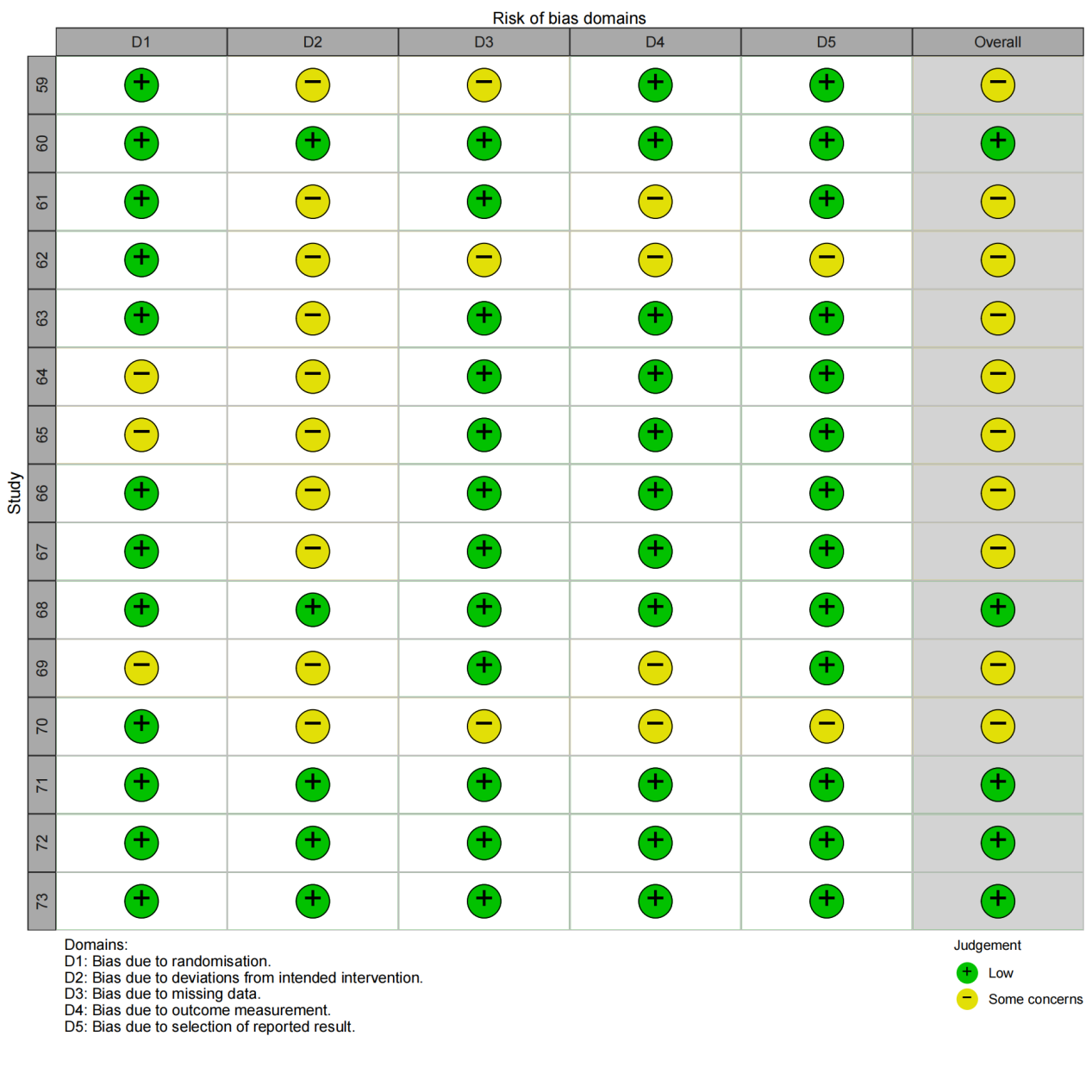


eFigure 9d Traffic lights summary of References 59~73
